# Gain and loss of an intron in a protein-coding gene in Archaea: the case of an archaeal RNA pseudouridine synthase gene

**DOI:** 10.1186/1471-2148-9-198

**Published:** 2009-08-11

**Authors:** Shin-ichi Yokobori, Takashi Itoh, Shigeo Yoshinari, Norimichi Nomura, Yoshihiko Sako, Akihiko Yamagishi, Tairo Oshima, Kiyoshi Kita, Yoh-ichi Watanabe

**Affiliations:** 1 Department of Molecular Biology, School of Life Science, Tokyo University of Pharmacy and Life Science, Horinouchi, Hachioji, Tokyo 192-0392, Japan; 2 Japan Collection of Microorganisms, RIKEN (The Institute of Physical and Chemical Research) BioResource Center, Wako, Saitama 351-0198, Japan; 3 Department of Biomedical Chemistry, Graduate School of Medicine, University of Tokyo, Bunkyo-ku, Tokyo 113-0033, Japan; 4 Division of Applied Biosciences, Graduate School of Agriculture, Kyoto University, Kyoto, Kyoto 606-8502, Japan; 5 Institute of Environmental Microbiology, Kyowa Kako, Tadao, Machida, Tokyo 194-0035, Japan

## Abstract

**Background:**

We previously found the first examples of splicing of archaeal pre-mRNAs for homologs of the eukaryotic CBF5 protein (also known as dyskerin in humans) in *Aeropyrum pernix, Sulfolobus solfataricus, S. tokodaii*, and *S. acidocaldarirus*, and also showed that crenarchaeal species in orders Desulfurococcales and Sulfolobales, except for *Hyperthermus butylicus, Pyrodictium occultum, Pyrolobus fumarii*, and *Ignicoccus islandicus*, contain the (putative) *cbf5 *intron. However, the exact timing of the intron insertion was not determined and verification of the putative secondary loss of the intron in some lineages was not performed.

**Results:**

In the present study, we determined approximately two-thirds of the entire coding region of crenarchaeal Cbf5 sequences from 43 species. A phylogenetic analysis of our data and information from the available genome sequences suggested that the (putative) *cbf5 *intron existed in the common ancestor of the orders Desulfurococcales and Sulfolobales and that probably at least two independent lineages in the order Desulfurococcales lost the (putative) intron.

**Conclusion:**

This finding is the first observation of a lineage-specific loss of a pre-mRNA intron in Archaea. As the insertion or deletion of introns in protein-coding genes in Archaea has not yet been seriously considered, our finding suggests the possible difficulty of accurately and completely predicting protein-coding genes in Archaea.

## Background

Introns in protein-coding genes and pre-mRNA splicing are ubiquitous in Eukarya and, to a lesser extent, in Bacteria. Until 2001, pre-mRNA splicing had not been reported in Archaea. In 2002, we reported the first examples of archaeal pre-mRNA splicing for homologs of the eukaryotic CBF5 (centromere binding factor 5 in yeast, or dyskerin in humans) protein in *Aeropyrum pernix, Sulfolobus solfataricus*, and *S. tokodaii *[[1], also in *S. acidocaldarius*, see [2] in 2006]. We found that the cleavage of the pre-mRNA depends on the recognition of a bulge-helix-bulge (BHB)-like structure in the precursor [1,2] by the splicing endonuclease EndA [3]. In Archaea, pre-tRNA and pre-rRNA splicings also depend on the same system [[4,5]; reviewed in [1]]. Although most species from the orders Desulfurococcales and Sulfolobales have the (putative) *cbf5 *intron, *H. butylicus, P. occultum, P. fumarii*, and *I. islandicus *in the order Desulfurococcales do not contain the intron [1,2]. This observation suggested putative secondary loss of the intron. However, phylogenetic analysis of the Cbf5 protein sequences did not resolve the relationships between species from different orders of Crenarchaeota, likely due to the short sequence (about 70 amino acid residues) studied in the analysis [2].

In the present study, we determined a formerly undetermined region of *cbf5 *sequences from the previously characterized 27 species and new sequences from an additional 16 species. We studied 43 species, which were almost all the available species from type culture collections. We determined up to two-thirds of the coding region, corresponding to about 220 amino acid residues, and then examined the timing of the gain and the possible loss of the intron in the archaeal protein-coding gene. We found that the intron existed in the *cbf5 *gene in the common ancestor of the orders Desulfurococcales and Sulfolobales, and then the intron was lost in some lineages in the order Desulfurococcales.

## Methods

### Strains and DNA for PCR screening

Most crenarchaeal strains were grown according to the conditions suggested by the Japan Collection of Microorganisms (JCM) [2]. Some strains were purchased from the German Collection of Microorganisms and Cell Cultures (DSMZ). In most PCR reactions, the crude DNA was prepared as described previously [2]. In the case of *Thermofilum pendens*, the obtained DNA was too dilute; thus, for PCR with degenerate primers at the initial screening, the DNA was pre-amplified by using the illustra GenomiPhi DNA Amplification Kit (GE Healthcare Bioscience, Shinjuku, Tokyo, Japan). The DNA of '*Caldococcus noboribetus' *was kindly provided by Dr. M. Aoshima (University of Tokyo). The DNA from *Aeropyrum pernix *strains was prepared as previously described [6]. See [Additional file 1], Table 1 and 2 (for *Aeropyrum pernix *strains) for further information about the strains.

**Table 1:** Strains and size of cbf5 intron.

order*	family**	species***	references****	intron (bp)
D	D	*Acidilobus aceticus*	[2],a	29
D	D	*Aeropyrum camini*	[2],a	37
D	D	*Aeropyrum pernix*	[1,2,52]	38
D	D	*Caldisphaera lagunensis*	[2],a	29
D	D	*Desulfurococcus amylolyticus*	a	21
D	D	*Desulfurococcus mobilis*	[2],a	16
D	D	*Desulfurococcus mucosus*	a	16
D	D	*Ignicoccus hospitalis*	[31]	0
D	D	*Ignicoccus islandicus*	[2],a	0
D	D	*Ignicoccus pacificus*	a	0
D	D	*Ignisphaera aggregans*	a	39
D	D	*Staphylothermus hellenicus*	a	36
D	D	*Staphylothermus marinus*	[2],a	36
D	D	*Stetteria hydrogenophila*	[2],a	33
D	D	*Sulfophobococcus zilligii*	[2],a	32
D	D	*Thermodiscus maritimus*	[2],a	44
D	D	*Thermosphaera aggregans*	[2],a	19
D	P	*Hyperthermus butylicus*	[2],a	0
D	P	*Pyrodictium abyssi*	a	0
D	P	*Pyrodictium brockii*	a	0
D	P	*Pyrodictium occultum*	[2],a	0
D	P	*Pyrolobus fumarii*	[2],a	0
D	u	*'Caldococcus noboribetus'*	a	29
S	S	*Acidianus ambivalens*	a	20
S	S	*Acidianus brierleyi*	a	19
S	S	*Acidianus infernus*	[2],a	20
S	S	*Metallosphaera hakonensis*	[2],a	19
S	S	*Metallosphaera sedula*	[2],a	19
S	S	*Stygiolobus azoricus*	[2],a	22
S	S	*Sulfolobus acidocaldarius*	[2,48]	22
S	S	*Sulfolobus acidocaldarius*	[2],a	22
S	S	*Sulfolobus metallicus*	[2],a	22
S	S	*Sulfolobus shibatae*	[2],a	23
S	S	*Sulfolobus solfataricus*	[1,53]	22
S	S	*Sulfolobus tokodaii*	[1,54]	31
S	S	*Sulfurisphaera ohwakuensis*	[2],a	31
T	Tf	*'Thermofilum librum'*	a	0
T	Tf	*Thermofilum pendens*	a	0
T	Tp	*Caldivirga maquilingensis*	[2],a	0
T	Tp	*Pyrobaculum aerophilum*	[55]	0
T	Tp	*Pyrobaculum arsenaticum*	a	0
T	Tp	*Pyrobaculum islandicum*	a	0
T	Tp	*Pyrobaculum oguniense*	[2],a	0
T	Tp	*Pyrobaculum organotrophum*	a	0
T	Tp	*Pyrobaculum calidifontis*	unpublished	0
T	Tp	*Thermocladium modestius*	[2],a	0
T	Tp	*Thermoproteus neutrophilus*	[2],a	0
T	Tp	*Thermoproteus tenax*	[2],a	0
T	Tp	*Vulcanisaeta distributa*	[2],a	0
T	Tp	*Vulcanisaeta souniana*	a	0
C	C	*'Cenarchaeum symbiosum'*	[2,56]	0
N	N	*'Nitrosopumilus maritimus'*	unpublished	0
K		*Ca*. Korarchaeum cryptofilum	[30]	0

*: D; Desulfurococcales, S; Sulfolobales, T; Thermoproteales, C; 'Cenarchaeales', N; 'Nitrosopumilales', K; 'Korarcheaota' (phylum)

**: D; Desulfurococcaceae, P; Pyrodictiaceae, u; unclassified, S; Sulfolobaceae, Tf; Thermofilaceae, Tp; Thermoproteaceae, C; Cenarchaeaceae, N; 'Nitrosopumilaceae'

***: Ca., Candidatus

****: Only the references for the data used in the present study are shown. See the text in the detail. a; the present study.

**Table 2:** Introns in *cbf5 *and in rRNA genes in *Aeropyrum pernix *strains

Strain	cbf5	arnS#1*	arnS#2*	arnL#3*	arnL#4*
K1	type 1	Ialpha		Ibeta	Igamma
OH1	type 2				
OH2	type 2				
OH3	type 1				
TB1	type 1	Idelta	Iepsilon	Ibeta	
TB2	type 1	Idelta	Iepsilon	Ibeta	
TB3	type 1	Idelta	Iepsilon	Ibeta	
TB4	type 2	Idelta	Iepsilon	Ibeta	Izeta
TB5	type 1	Idelta	Iepsilon	Ibeta	Izeta
TB6	type 1	Idelta	Iepsilon	Ibeta	Izeta
TB7	type 1	Idelta	Iepsilon	Ibeta	Izeta
TB8	type 1	Idelta	Iepsilon	Ibeta	Igamma

*, positions and type of introns were designated as in [6].

### PCR screening of archaeal cbf5 genes

The typical reaction mixture for PCR (25 μl) contained 1× reaction buffer (Takara Bio, Ohtsu, Shiga, Japan), 0.2 mM of each deoxynucleoside triphosphate, 0.5 μl of template, and 2.5 units of ExTaq (Takara Bio). At the first screening to obtain the gene fragment between Gly57 and Ile143 (*Sulfolobus tokodaii *numbering) with M13 sequencing primer (P-486 and P-583) binding sites at both ends, we used a set of degenerate primers based on conserved regions among known crenarchaeal Cbf5 sequences (1 μM each of P-1607 and P-1608 (forward), and 2 μM P-1516 (reverse)). For *Ignicoccus pacificus, Staphylothermus hellenicus, Pyrodictium brockii, 'Caldococcus noboribetus'*, and *Ignisphaera aggregans*, 2 μM P-1608 was used as a forward primer instead of the combination of P-1607 and P-1608 to improve the amplification efficiency. In the case of *Pyrobaculum arsenaticum, P. islandicum*, and *P. organotrophum*, 2 μM P-1911, specifically designed for the *Pyrobaculum *species, was used as the forward primer. The PCR products were purified and sequenced as described previously [2].

To obtain additional sequence information from the 3' region of the gene in the species described above as well as in the species that we previously studied [2], we designed degenerate primers P-1609 and P-1610 with M13 sequencing primer binding sites and performed semi-nested PCR with two species-specific primers (forward) and P-1609/P-1610 (reverse). The second PCR products, or in some cases the first PCR products, if observed, were purified and sequenced with specific PCR primers or the universal reverse primer (as mentioned above). If necessary, internal primers were designed and used in primer walking.

In the case of *Sulfolobus metallicus*, the reverse primer hybridized outside of the *cbf5 *gene in the 3' downstream region, and the PCR product included up to the termination codon of the *cbf5 *gene as well as the partial sequence of another coding region that partially overlapped *cbf5*.

In the initial screening of the *Thermofilum *species, the above-mentioned combinations of primers did not work. Thus, we used P-1835 (forward) and P-1838 (reverse). Only the *T. pendens *pre-amplified DNA gave a product with the expected size. Sequence information from the product was used to design specific primers (P-1856 and P-1857). A semi-nested PCR that used P-1856 (in the first reaction, forward) and P-1857 (in the second reaction, forward) and a degenerate primer (P-1610, reverse) gave the products from non-amplified DNAs from both *T. pendens *and '*Thermofilum librum'*. Using the obtained sequence information, we designed specific primers (P-1860 and P-1862). To amplify the remaining portion of the 5' region of '*T. librum' cbf5*, semi-nested PCR that used P-1608 (forward) and P-1862 (in the first reaction) and P-1680 (in the second reaction) was performed.

Primer sequences as well as species-specific primers used in the nested PCR and sequencing analysis are shown in Table 3 and [Additional file 2], respectively. The deduced protein sequences from the *Thermofilum *species are identical; thus, we used only one sequence designated as *Thermofilum *in the phylogenetic analysis.

**Table 3:** Oligonucleotides

name	sequence (5' to 3')*	target (peptide sequence)**
P-486	GAGCGGATAACAATTTCACACAGG	pUC/M13 rv
P-517	CCTACCCCATGAGAGGCCGTTGGA	A. pernix, fw
P-518	GGCCTATGGAGCTGCATCACGCA	A. pernix, rv
P-583	GTTTTCCCAGTCACGACGTTGTA	pUC/M13 fw
P-1516	gagcggataacaatttcacacaggaVKGGKGGYYTYTGRTADAT	cbf5, rv (IYQ(K/R)PP(L/V))
P-1607	gttttcccagtcacgacgttgtaGGKCCKACKTCKCAYGARGT	cbf5, fw (GPTSHEV)
P-1608	gttttcccagtcacgacgttgtaGGKCCKACKAGYCAYGARGT	cbf5, fw (GPTSHEV)
P-1609	gagcggataacaatttcacacaggARYTCKCCYTTNAGNGT	cbf5, rv (TLKGEL)
P-1610	gagcggataacaatttcacacaggARYTCKCCYTTYAANGT	cbf5, rv (TLKGEL)
P-1835	gttttcccagtcacgacgttgtaGGKCCKACNAGYCAYGA	cbf5 fw (GPTSHE)
P-1838	gagcggataacaatttcacacaggTKGGRTCNAGNGTNCC	cbf5 rv (TTLDP(K/N/R))
P-1856	GGTTGTAGCGTGGCTTAGGAAGCT	T. pendens, fw
P-1857	GCTCCTAGGGATAGAGAGAATAGC	T. pendens, fw
P-1860	TCGAACCTCCCTCTTCACAGCAGA	Thermofilum, rv
P-1862	CAGCTTCGCACCAGACATGGAGGA	Thermofilum, rv
P-1911	gttttcccagtcacgacgttgtaGGKCCKAGYAGYCAYGA	cbf5, fw (GPSSHE)

*; sequencing primer binding site is shown in lower case.

**; fw; forward, rv; reverse

K = G or T; R = G or A; Y = T or C; N = T, C, G or A; D = T, G, or A; V = C, G or A; S = C or G.

For strains of *Aeropyrum pernix*, PCR was performed with P-517 and P-518 as described in [1]. The PCR product was treated with SAP-IT (GE Healthcare Bioscience) and used directly (without cloning) in a sequencing reaction with one of the PCR primers to determine a 249-bp region.

Newly reported sequences were deposited in the DDBJ/EMBL/GenBank database under the accession numbers [DDBJ:AB245528] to [DDBJ:AB245554], [DDBJ:AB261609] to [DDBJ:AB261610], [DDBJ:AB304834] to [DDBJ:AB304847], and [DDBJ:AB469400] to [DDBJ:AB469410].

During the preparation of this manuscript, genome sequence data from *Staphylothermus marinus *[7] (release date, Feburary 21, 2007), *Hyperthermus butylicus *[8] (release date; January 22, 2007), *Metallosphaera sedula *[Genbank:CP000682] (released date; June 30, 2008), *Thermofilum pendens *[9] (release date; December 18, 2006), *Caldivirga maquilingensis *[Genbank: CP000852] (release date: October 5, 2007), *Pyrobaculum arsenaticum *[Genbank: CP000660] (release date; November 1, 2007), *Pyrobaculum islandicum *[Genbank: CP000504] (release date; November 1, 2007), and *Thermoproteus neutrophilus *[Genbank: CP001014] (release data; March 27, 2008), of which *cbf5 *we sequenced, became available. However, the gene annotation was different from ours when the gene had the putative intron (see below). Our sequence determination was independently performed before the release date of the data from other groups; the data from the additional 16 species were deposited to the database on May 31, 2007. Note that, as for *S. marinus *and *H. butylicus*, we released the partial *cbf5 *sequence data on June 28, 2006. Thus, we used our data for the above-mentioned seven species in the following analysis. To avoid the confusion, we did not include information of the above-mentioned seven species from other groups in Table 1.

### Sequence and phylogenetic analysis

**Figure 1:**
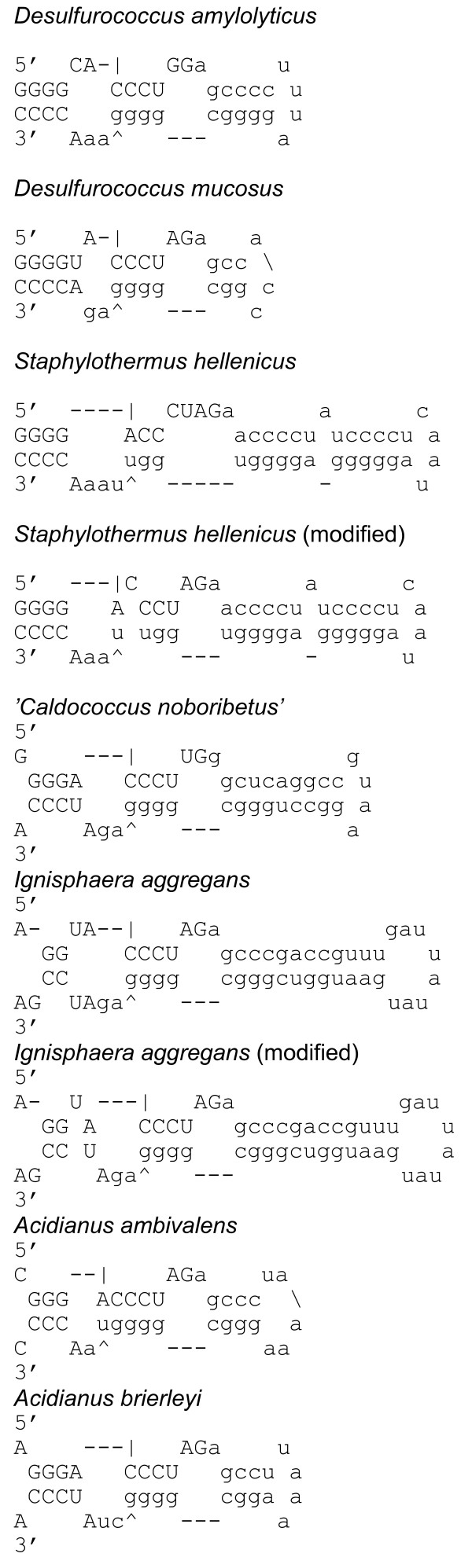
**Secondary structures of (putative) exon-intron boundaries of crenarchaeal *cbf5 *newly identified in this study**. The structures were predicted with mfold [10,11]. In the cases of *Staphylothermus hellenicus *and *Ignisphaera aggregans*, manually modified structures are also shown. The predicted exons and introns are shown in upper and lower cases, respectively.

**Figure 2:**
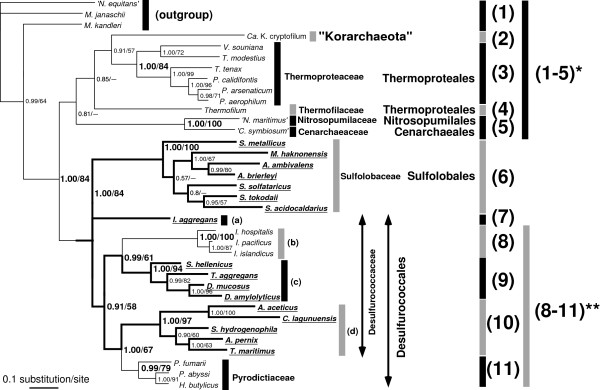
**Bayesian phylogenetic tree of representative Cbf5 protein sequences**. Thirty-six species were selected for tree reconstruction and were divided into 11 categories. See sequence details in [Additional file 1], except for *Methanocaldococcus jannaschii *[Genbank:AAB98132], '*Nanoarchaeum equitans' *[Genbank:AAR39298)], and *Methanopyrus kandleri *[Genbank:AAM01350] as the outgroups. To analyze the monophyletic status of orders Desulfurococcales + Sulfolobales (analysis 1), categories 8 to 11 were treated as a single category. To analyze the interrelationship within Desulfurococcales (analysis 2), categories 1 to 5 were treated as a single category. Posterior probability (PP) for Bayesian Inference and bootstrap probability (BP; %) for the maximum likelihood method are shown at the nodes. Bold lines show lineages with the (putative) *cbf5 *intron.

RNA secondary structure was predicted with the mfold version 3.1 web server (Figure 1) [10,11]. The putative exon-intron boundaries were assigned between the first and second letters of the codon for the catalytic aspartic residue of Cbf5 [1]. The predicted BHB motifs were also considered for the prediction of the exon-intron borders (Figure 1). The alignment of the cbf5 protein sequences (56 operational taxonomic units (OTUs)) was performed with ClustalW [12] (Additional file 3). Well-aligned regions were then selected (201 sites in total) with Gblocks [13] with the following parameters: the minimum number of sequences for a conserved position was 29, the minimum number of sequences for a flanking position was 47, the maximum number of contiguous nonconserved positions was 10, and the minimum length of a block was 5. Tree reconstruction was performed with the Treefinder version of June 2008 (for maximum likelihood inference) [14] under the WAG+G model (WAG model [15] with consideration of gamma-shaped rate variation (4-parameter model) [16]) and MrBayes 3.12 (for Bayesian inference) [17] under the WAG+I+G model (WAG model with consideration of gamma-shaped rate variation (4-parameter model) and a proportion of invariable sites). For the Bayesian inference analysis (Figures 2 and Additional file 4), a Markov chain Monte Carlo analysis was run for 2,000,000 generations, and trees were built in 100-generation intervals (burn-in = 5,000). Statistical support for the maximum likelihood inference tree was evaluated with a non-parametric bootstrap test with 1,000 re-sampling events. The AU (approximately unbiased) [18], NP (non-scaled bootstrap probability) [19], and KH (Kishio-Hasegawa) [20] tests were performed with CONSEL [21]. For these tests, to reduce number of trees to be considered, analyses were performed with the grouping of the sequences to form a reduced number of the dataset (36 OTUs, 202 sites) with Codeml in PAML 3.13 [22] under the WAG+G model (Additional file 5, see below) (for the alignment, see Additional file 6). The tree topologies tested were selected by the preliminary maximum likelihood analysis performed with TREE-PUZZLE 5.2 [23] (Figure 2). Same dataset was also used for Bayesian inference with MrBayes 3.12. In Figure 2, the obtained tree with Bayesian inference was shown. The 16S rRNA phylogenetic tree was reconstructed by using Treefinder version of June 2008 and MrBayes 3.12 under the GTR+I+G model (GTR: general time reversible, 6-parameter model). The 16S rRNA gene sequences (49 OTUs) were aligned with Clustal X [24] under the default condition. The well-aligned regions were selected (1,122 sites in total) with Gblocks under the default condition for nucleotide sequences. The model was selected by using modeltest 3.7 [25] with PAUP4b10 [26] under Akaike's Information Criterion. The alignment of the *cbf5 *intron with the flanking sequences was performed with R-coffee [27] using default parameters. Most calculations were performed using a MacPro (Apple) with a 3.0-GHz 8-core (4 × 2) Xeon Intel processor and 8-GB memory.

## Results and discussion

Our previous analysis of crenarchaeal *cbf5 *genes showed that only orders Desulfurococcales and Sulfolobales have the (putative) intron in their *cbf5 *genes, although some species in the order Desulfurococcales do not have the intron. However, phylogenetic analysis with the previous dataset did not strongly support the sister grouping of orders Desulfurococcales and Sulfolobales without species from other orders, and the phylogenetic positions of the species in Desulfurococcales, which do not have the intron, were unclear [2].

To improve the phylogenetic analysis of the *cbf5 *gene, we extended the analyzed region of the genes from 27 species to include an additional area in the 3' region (from about 70 to 220 amino acid residues), and we added new sequences from an additional 16 crenarchaeal species. We also added the recent information from the newly determined crenarchaeal and korarchaeal genomes. The species and the intron size information are summarized in [Additional file 1]. When the presence of the intron was expected, the new putative exon-intron borders from seven species among the additional 16 species were subjected to a prediction of their secondary structures (Figure 1. For 18 species which have the (putative) intron among the previously characterized 27 species, see reference [2]). Except for the cases of *'Caldococcus noboribetus' *and *Acidianus brierleyi*, the predicted structures in the pre-mRNAs have an unconventional BHB structure [28], which should be recognized and cleaved by the hetero-oligomeric splicing endonuclease, as demonstrated previously [2]. Recent X-ray crystallography has revealed that hetero-oligomeric splicing endonuclease is a dimer of hetero-dimers [29]. The predicted cleavage sites between the second and the third residues in the bulges of the BHB motif were consistent with the expected exon-intron borders, suggesting that the predicted exon-intron borders were convincing. In fact, partial cDNA sequences of spliced *cbf5 *mRNA from *Desulfurococcus amylolyticus*, *Desulfurococcus mucosus*, *Staphylothermus hellenicus, Acidianus brierleyi *and *Ignisphaera aggregans *were consistent with the predictions (Watanabe, Y. and Itoh, T. unpublished results), although the definite identification of the borders of the remaining species requires a cDNA sequencing and cleavage study using splicing endonuclease. Results from our present study, together with the previous study [2], indicate that among the order Desulfurococcales, *Ignicoccus *spp. and all species from family Pyrodictiaceae do not have the *cbf5 *intron.

Using a new dataset, we reconstructed phylogenetic trees of the cbf5 protein sequence by using maximum likelihood (not shown) and Bayesian methods [Additional file 4]. These trees suggested the monophyly of the cbf5 protein sequences from orders Desulfurococcales and Sulfolobales. We verified this monophyly with several statistical tests (analysis 1, [Additional file 5]). To finish the computation within a reasonable time (approximately 1 week) using the available computational environment with a reduced number of trees to be considered, we first reduced the number of sequences in the dataset and reconstructed the phylogenetic tree (Figure 2). There was no significant difference in the tree topology before and after the reduction of the sequence (compare [Additional file 4] and Figure 2). Then, we fixed the relationships within each of the eight groups (Figure 2) and examined the relationships between the groups (analysis 1, Additional file 5). The results of the tests supported the monophyly of the sequences from orders Desulfurococcales and Sulfolobales (AU; P = 0.938, NP; P = 0.799, KH; P = 0.907) and also suggested the inclusion of the sequence of 'Korarchaeum' into the crenarchaeal sequences. The result is consistent with the phylogenetic association of rRNA and protein sequences from 'Korarchaeum' and Crenarchaea [30].

The sequences from the species of family Pyrodictiaceae and *Ignicoccus *spp. are grouped independently, and these monophylies were strongly supported with high statistical values in the trees (Figure 2, see also [Additional files 5 and 6]). Although among orders Desulfurococcales and Sulfolobales, these groups are not likely to be the earliest branching (Figure 2, see also [Additional file 4]), the branching order among order Desulfurococcales, particularly of *Ignisphaera aggregans*, was uncertain. Thus, we examined whether the sequences of family Pyrodictiaceae and/or *Ignicoccus *spp. branched earliest among the order Desulfurococcales, except for *Ignisphaera aggregans*, by using AU, NP, and KH tests of an alternative grouping set (analysis 2, Figure 2, [Additional file 7]). The monophyly of the Desulfurococcaceae (i.e., the earliest branching of the Pyrodictiaceae sequence) was rejected by the AU test (P = 0.029) and NP test (P = 0.001) (95% significance level) but not by the KH test (P = 0.075). If *Ignisphaera aggregans *was not considered, the monophyly of the Desulfurococcaceae (excluding *Ignisphaera aggregans *and Pyrodictiacean species) would be supported by only small probabilities by the AU test and KH test (P = 0.062, and 0.071, respectively) and rejected by the NP test (P < 0.001). The monophyletic grouping of the Desulfurococcaceae (group d in Figure 2) with the intron and the Pyrodictiaceae was supported by the AU, NP, and KH tests (P = 0.831, 0.697, and 0.829, respectively). These results suggest that the sequences of the Pyrodictiaceae (as seen in the Bayesian tree of Figure 2) are unlikely to be the earliest branching. The monophyletic grouping of Desulfurococcaceae (c) with the intron and *Ignicoccus *spp. (as seen in the tree of Figure 2) was also supported by the AU, NP, and KH tests (P = 0.82, 0.605, and 0.78, respectively). These results also suggest that the sequence of *Ignicoccus *spp. is not likely to be the earliest branching as seen in the Bayesian tree of Figure 2. The monophyly of Desulfurococcaceae (b) + Desulfurococcaceae (c) (appeared in the Bayesian tree of Figure 2) could not be rejected by the AU and KH tests because of their medium probabilities (P = 0.155 and 0.187, respectively), but this monophyly was rejected by the NP test (P = 0.02). The monophyly of *Ignisphaera aggregans *+ Pyrodictiaceae also cannot be rejected by the AU, NP, and KH tests because of their medium probabilities (P = 0.313, 0.212, and 0.219, respectively). The monophyly of Desulfurococcaceae (c and d) + Pyrodictiaceae was not rejected by the tests (AU; P = 0.287, NP; P = 0.078, KH; P = 0.163). Finally, the monophyly of species with the intron was not rejected by the tests (AU; P = 0.329, NP; P = 0.058, KH; P = 0.194). Therefore, the sequence of both *Ignicoccus *spp. and the Pyrodictiaceae was unlikely to be the earliest simultaneous branching, as seen in the tree presented in Figure 2. These results suggest that the sequences of these groups are not likely to be the earliest branching, although the possibility was not completely excluded. As a reference, we constructed a phylogenetic tree of 16S rRNA of the corresponding species by using the Bayesian method [Additional file 8]. The 16S rRNA tree also supported the monophyletic groupings of orders Desulfurococcales and Sulfolobales, *Ignicoccus *spp. and Desulfurococcaceae (c), and Pyrodictiaceae and Desulfurococcaceae (d), suggesting that there was no obvious gene transfer of *cbf5 *from outside of orders Desulfurococcales and Sulfolobales. About 6% of protein-coding genes in *Ignicoccus hospitalis *are thought to be transferred from its symbiont *'Nanoarchaea' *[31]. However, in our analysis, the monophyletic grouping of *cbf5 *genes in *Ignicoccus spp*. with the nanoarchaeal sequence was not supported. Thus, the *cbf5 *gene in *Ignicoccus spp*. is not likely due to gene transfer of the intron-less nanoarchaeal *cbf5 *gene.

**Figure 3:**
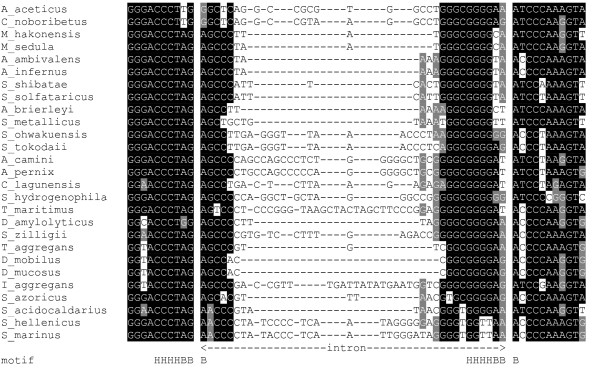
**Alignment of *cbf5 *introns with their flanking sequences**. The data was shaded by using the Boxshade server [57]. Residues conserved among more than 50% of the sequences are shown on black background. Residues similar to the conserved residue, or conserved among purines (or pyrimidines), are shown on gray background. The intron region and the region corresponding to the BHB motif (bulge as B, helix as H) are also shown.

We also aligned the (putative) *cbf5 *introns with the flanking sequences using the program R-coffee with the RNA secondary structure prediction option (Figure 3). The alignment showed some conservation in the intron region beyond base-pairing with the exon regions to maintain the motif required for cleavage by the splicing endonuclease, suggesting a common origin for these introns. Note that the internal region of the introns was highly variable likely due to the independence of recognition by the splicing endonuclease during the cleavage at the exon-intron borders.

The origin of the archaeal *cbf5 *intron is still unclear. We previously proposed that relaxed substrate specificity [2,32-34] of the hetero-oligomeric splicing endonuclease [3,35] led to the birth of the pre-mRNA intron, which frequently contains the relaxed cleavage motif ([2] and this study). In particular, the recognition of the relaxed cleavage motif within a non-tRNA context has been shown to be characteristic of crenarchaeal hetero-tetrameric splicing endonuclease [2,29,32,33]. Although the intron sizes in cbf5 and rRNA are different from one another, as discussed below, archaeal rRNA introns are observed mainly in crenarchaeal species, which are expected to have the crenarchaeal hetero-tetrameric splicing endonuclease [36]. In some cases, archaeal rRNA introns also have the relaxed cleavage motifs [37]. The size of archaeal tRNA introns (11 to 175 nucleotides) are more similar to those in crenarchaeal *cbf5*, and accumulation of tRNA introns in crenarchaeal species is observed [36]. The unconventional cleavage motif at the exon-intron borders and the intron location at the position rather than the usual position "37/38" of tRNA intron are also observed more frequently in crenarchaeal species [28,36]. The contribution of the hetero-tetrameric splicing endonuclease is suggested for the cleavage of the unconventional motif, and has been demonstrated by the crenarchaeal hetero-tetrameric splicing endonuclease (reviewed in [29]).

**Figure 4:**
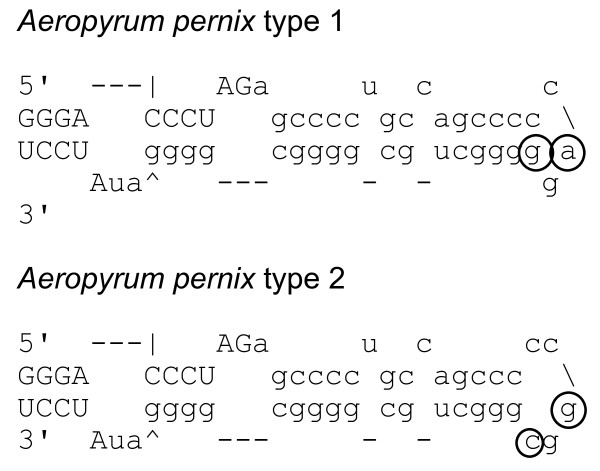
**Two types of exon-intron boundaries of *Aeropyrum pernix cbf5***. The exons and introns are shown as in Figure 1. Residues substituted between each type are circled.

Numerous archaeal rRNA introns contain the open reading frame for DNA endonuclease, which functions as a homing endonuclease to make the intron as a mobile element (reviewed in [38]). Apparently, the archaeal *cbf5 *intron is too short (from 16 to 44 bp, see [Additional file 1]) to encode such a nuclease. Nomura et al. found that *A. pernix *isolates have variations in the number, sequence, and positions of rRNA introns [6] (see also Table 2). In the present study, we determined partial *cbf5 *sequences of these *A. pernix *isolates. Together with the results of the previous studies for type strain K1 [1], we found that at the corresponding positions, all of the analyzed *cbf5 *genes have a putative intron, classified as type 1 or type 2 (Figure 4, the distribution is mentioned in Table 2), which contains only two base substitutions. There was no correlation between the variation of *cbf5 *and rRNA introns (Table 2). Although sequence variation of rRNA introns between *A. pernix *isolates (one to two substitutions in I beta or one substitution in I epsilon) were observed, this was not correlated with the variation of the *cbf5 *intron. However, a correlation between the *cbf5 *intron and *radA *phylogeny shown by Nomura et al. [6] was observed (not shown). Our results show that, as for the large-scale in-del event, the *cbf5 *intron was more conserved than the rRNA introns with the homing DNA endonuclease gene. However, Nomura et al. [6] also found that some of the rRNA introns are deletion derivatives of the introns with an open reading frame. For example, *A. perinix *introns I delta and I zeta are deletion derivatives of I alpha and I gamma, respectively [6]. The contemporary *cbf5 *introns may be examples of such deletion derivatives. Proof of this possibility requires further taxonomic sampling of *cbf5 *genes to find the intron that includes the protein-coding sequence.

Peng et al. showed that during the generation of infection, putative 12-bp introns were inserted into protein-coding genes in an archaeal virus genome, although splicing was not demonstrated and the mechanism of insertion of the 12-bp sequence is unknown [39]. Interestingly, the sizes of the *cbf5 *introns from *Staphylothermus hellenicus *and *S. marinus *are 36 bp (3 times 12); thus, mechanisms of insertion of archaeal *cbf5 *introns and the putative introns in the archaeal virus genome may be related. Furthermore, the *cbf5 *introns of *Stetteria hydrogenophila *(33 bp) and *Ignisphaera aggregans *(39 bp), as well as *S. hellenicus *and *S. marinus*, do not change the reading frame. The putative introns in the virus genome may not be spliced out and the coding region with such insertions may produce functional proteins. However, in the case of *cbf5 *introns, the insertion disrupts the codon of the catalytic residue of the protein [1,40], and thus these must be spliced out if the organism needs the functional protein.

One possible explanation of the putative secondary loss of the *cbf5 *intron in certain lineages is that the intron-containing gene is replaced with a sequence without the intron, possibly produced by reverse transcription of the spliced mRNA [41], or the spliced mRNA itself. Although reverse transcriptase activity has not been observed in crenarchaeal cells, the presence of a putative reverse transcriptase gene in some archaeal genomes has been suggested [42]. In fact, in the sequenced genomes of *Ignicoccus hospitalis *and *Hyperthermus butylicus *(family Pyrodictiaceae) with the putative secondary loss of the *cbf5 *intron, candidate reverse transcriptase genes were identified [Additional files 9 and 10]. An alternative possibility could be the requirement of higher activity of pseudouridine synthase in a certain environment. Previously, we proposed that the *cbf5 *intron functions as a negative regulator of the expression of pseudouridine synthase [1]. Archaeal Cbf5 catalyzes pseudouridine formation in rRNA and tRNA together with other associated proteins using a guide RNA [40,43] or without a guide RNA [44]. Incorporation of pseudouridine in RNA increases the thermodynamic stability of RNA [45]. Furthermore, pseudouridylation of tRNA at position 55 by TruB in mesophilic bacteria *Escherichia coli *supports the resistance to higher temperature [46]. Archaeal Cbf5, a member of truB family [47], also forms a pseudouridine in tRNA at position 55 [44]. Thus, at extremely high temperatures, the organisms might not prefer the down-regulation system of the pseudouridine synthase and lose it.

## Conclusion

The results of the present study suggest that *cbf5 *gained the intron in the common ancestor of orders Desulfurococcales and Sulfolobales, and that *cbf5 *lost the intron independently in the ancestors of the family Pyrodictiaceae and *Ignicoccus *spp. Since we found the first examples of *cbf5 *introns, sequences of three crenarchaeal genomes with the *cbf5 *intron have been determined. However, the *cbf5 *intron in these genomes was misidentified (*S. acidocaldarius*; [48], see [2]) or ignored (*Staphylothermus marinus *[7], *Metallosphaera sedula*, [Genbank:CP000682]). Even for the first three examples in *A. pernix*, *S. solfataricus*, and *S. tokodaii*, the gene prediction of these examples was still confused with cases of translational frame-shifting by other researchers [49]. Although there was no confirmation of archaeal pre-mRNA splicing for genes other than *cbf5*, the presence of the putative intron in other protein-coding genes was predicted [39,50]. To completely understand protein-coding genes in archaeal genomes, tools for effective prediction of introns in archaeal protein-coding genes must be developed with comparative or computational methods [50,51]. Experimental confirmation of the predictions, including the putative *cbf5 *introns predicted in our studies, is indispensable.

## Authors' contributions

YW conceived the study and participated in its design, carried out the molecular genetic studies, participated in the sequence alignment, and drafted the manuscript. S. Yokobori participated in the design of the study, the sequence alignment, performed the statistical analysis, and helped draft the manuscript. TI, S. Yoshinari, and NN carried out the molecular genetic studies and helped draft the manuscript. YS, AY, TO, and KK participated in the design and coordination of the study and helped draft the manuscript. All authors read and approved the final manuscript.

## Supplementary Material

Additional file 1 **Strains and size of cbf5 intron**. Details of the strains studied, including strain numbers, accession numbers, are shown. Click here for file

Additional file 2 **Oligodeoxynucleotides not listed in Table **2. Information of additional PCR and sequencing primers are shown. Click here for file

Additional file 3 **Alignment of archaeal Cbf5 sequences used in the analysis for Additional file **4. #; selected positions for the analysis. Click here for file

Additional file 4 **Bayesian phylogenetic tree of crenarchaeal Cbf5 protein**. Crenarchaeal Cbf5 sequences, which are not included in Figure 2, are included. Click here for file

Additional file 5 **The results of statistical tests of analysis 1**. Comparisons of statistical supports of each grouping concerning the phylogeny of the outgroups of Sulfolobales and Desulfurococcales. Click here for file

Additional file 6 **Alignment of archaeal Cbf5 sequences used in the analysis for Figure **2. #; selected positions for the analysis. Click here for file

Additional file 7 **The results of statistical tests of analysis 2**. Comparisons of statistical supports of each grouping concerning the phylogeny within Sulfolobales and Desulfurococcales. Click here for file

Additional file 8 **Bayesian phylogenetic tree of the crenarchaeal 16S rRNA**. This is for comparison with cbf5 tree. Click here for file

Additional file 9 **Alignment of COG1353 proteins**. *Sulfolobus solfataricus *SSO1991, a representative of COG1353 which was predicted as a putative reverse transcriptase, and the homologs from *Hyperthermus butylicus*, and *Ignicoccus hospitalis *are included. Click here for file

Additional file 10 **Figure legends for Additional files**. Legends for Additional files 3, 4, 6, 8 and 9 are shown. Click here for file
