# Supplementary material for: Gain and loss of an intron in a protein-coding gene in Archaea: the case of an archaeal RNA pseudouridine synthase gene
Source: BMC Evol Biol. 2009 Aug 11;9:198. doi: 10.1186/1471-2148-9-198 (PMC2738675; doi:10.1186/1471-2148-9-198)
Supplement: Additional file 1 — Strains and size of cbf5 intron. Details of the strains studied, including strain numbers, accession numbers, are shown. [file 1471-2148-9-198-S1.pdf]

Table 1. Strains and size of cbf5 intron

order*	family**	species***	JCM strain number	reference****	accession number*****	intron (bp)
D	D	*Acidilobus aceticus*	11320	2,a	[DDBJ:AB245528]	29
D	D	*Aeropyrum camini*	12091	2,a	[DDBJ:AB245529]	37
D	D	*Aeropyrum pernix*	9820	1,2,52	[DDBJ:AB245523] (c),[DDBJ:BA000002]	38
D	D	*Caldisphaera lagunensis*	11604	2,a	[DDBJ:AB245530]	29
D	D	*Desulfurococcus amylolyticus*	9188	a	[DDBJ:AB304834]	21
D	D	*Desulfurococcus mobilis*	9186	2,a	[DDBJ:AB245531]	16
D	D	*Desulfurococcus mucosus*	9187	a	[DDBJ:AB304835]	16
D	D	*Ignicoccus hospitalis*	DSM18386	31	[Genbank:CP000816]	0
D	D	*Ignicoccus islandicus*	DSM13165	2,a	[DDBJ:AB245532]	0
D	D	*Ignicoccus pacificus*	DSM13166	a	[DDBJ:AB304836]	0
D	D	*Ignisphaera aggregans*	13409	a	[DDBJ:AB304841]	39
D	D	*Staphylothermus hellenicus*	10830	a	[DDBJ:AB304837]	36
D	D	*Staphylothermus marinus*	9404	2,a	[DDBJ:AB245533]	36
D	D	*Stetteria hydrogenophila*	10135	2,a	[DDBJ:AB245534]	33
D	D	*Sulfophobococcus zilligii*	10309	2,a	[DDBJ:AB245535]	32
D	D	*Thermodiscus maritimus*	11597	2,a	[DDBJ:AB245536]	44
D	D	*Thermosphaera aggregans*	DSM11486	2,a	[DDBJ:AB245537]	19
D	P	*Hyperthermus butylicus*	9403	2,a	[DDBJ:AB245538]	0
D	P	*Pyrodictium abyssi*	9394	a	[DDBJ:AB304838]	0
D	P	*Pyrodictium brockii*	9392	a	[DDBJ:AB304839]	0
D	P	*Pyrodictium occultum*	9393	2,a	[DDBJ:AB245539]	0
D	P	*Pyrolobus fumarii*	DSM11204	2,a	[DDBJ:AB245540]	0
D	u	*'Caldococcus noboribetus'*	13516	a	[DDBJ:AB304840]	29
S	S	*Acidianus ambivalens*	9191	a	[DDBJ:AB304842]	20
S	S	*Acidianus brierleyi*	8954	a	[DDBJ:AB304843]	19
S	S	*Acidianus infernus*	8955	2,a	[DDBJ:AB245541]	20
S	S	*Metallosphaera hakonensis*	8857	2,a	[DDBJ:AB245542]	19
S	S	*Metallosphaera sedula*	9185	2,a	[DDBJ:AB245543]	19
S	S	*Stygiolobus azoricus*	9021	2,a	[DDBJ:AB245544]	22
S	S	*Sulfolobus acidocaldarius*	DSM639	2,48	[DDBJ:AB245526] (c), [Genbank:CP000077	22
S	S	*Sulfolobus acidocaldarius*	9063	2,a	[DDBJ:AB245527] (c), [DDBJ:AB245545]	22
S	S	*Sulfolobus metallicus*	9184	2,a	[DDBJ:AB245546]	22
S	S	*Sulfolobus shibatae*	8931	2,a	[DDBJ:AB245547]	23
S	S	*Sulfolobus solfataricus*	11322	1,53	[DDBJ:AB245525] (c), [EMBL:AE006641]	22
S	S	*Sulfolobus tokodaii*	10545	1,54	[DDBJ:AB245524](c), [DDBJ:BA000023]	31
S	S	*Sulfurisphaera ohwakuensis*	9065	2,a	[DDBJ:AB245548]	31
T	Tf	*'Thermofilum librum'*	DSM2337	a	[DDBJ:AB261610]	0
T	Tf	*Thermofilum pendens*	DSM2475	a	[DDBJ:AB261609]	0
T	Tp	*Caldivirga maquilingensis*	10307	2,a	[DDBJ:AB245549]	0
T	Tp	*Pyrobaculum aerophilum*	9630	55	[Genbank:AE009441]	0
T	Tp	*Pyrobaculum arsenaticum*	11321	a	[DDBJ:AB304844]	0
T	Tp	*Pyrobaculum islandicum*	9189	a	[DDBJ:AB304845]	0
T	Tp	*Pyrobaculum oguniense*	10595	2,a	[DDBJ:AB245550]	0
T	Tp	*Pyrobaculum organotrophum*	9190	a	[DDBJ:AB304846]	0
T	Tp	*Pyrobaculum calidifontis*	11548	unpublished	[Genbank:CP000561]	0
T	Tp	*Thermocladium modestius*	10088	2,a	[DDBJ:AB245551]	0
T	Tp	*Thermoproteus neutrophilus*	9278	2,a	[DDBJ:AB245552]	0
T	Tp	*Thermoproteus tenax*	9277	2,a	[DDBJ:AB245553]	0
T	Tp	*Vulcanisaeta distributa*	11212	2,a	[DDBJ:AB245554]	0
T	Tp	*Vulcanisaeta souniana*	11219	a	[DDBJ:AB304847]	0
C	C	*'Cenarchaeum symbiosum'*		2,56	[Genbank:DP000238]	0
N	N	*'Nitrosopumilus maritimus'*		unpublished	[Genbank:CP000866]	0
K		*Ca.* Korarchaeum cryptofilum		30	[Genbank:CP000968]	0

*: D; Desulfurococcales, S; Sulfolobales, T; Thermoproteales, C; 'Cenarchaeales', N; 'Nitrosopumilales', K; 'Korarcheaota' (phylum)

**: D; Desulfurococcaceae, P; Pyrodictiaceae, u; unclassified, S; Sulfolobaceae, Tf; Thermofilaceae, Tp; Thermoproteaceae, C; Cenarchaeaceae, N; 'Nitrosopumilaceae'

***: Ca., Candidatus

****: Only the references for the data used in the present study are shown. See the text in the detail. a; the present study.

*****: Only the number for the data used in the present study are show. (c), cDNA
