# Supplementary material for: Gain and loss of an intron in a protein-coding gene in Archaea: the case of an archaeal RNA pseudouridine synthase gene
Source: BMC Evol Biol. 2009 Aug 11;9:198. doi: 10.1186/1471-2148-9-198 (PMC2738675; doi:10.1186/1471-2148-9-198)
Supplement: Additional file 2 — Oligodeoxynucleotides not listed in Table 2. Information of additional PCR and sequencing primers are shown. [file 1471-2148-9-198-S2.pdf]

Additional file 2. Oligodeoxynucleotides not listed in Table 2.

name	sequence(5' to 3')	target*	purpose
P-1611	GTATGCGTAATGCAAGTCCACTGT	S. shibatae fw	PCR
P-1612	GGAGTACGTGTGCGTGATGCAGCT	A. camini fw	PCR, 2nd
P-1613	CTCAGATAAAGTCTACGTGGCTGT	T. neutrophilus fw	PCR, 1st
P-1616	TGTTGCATGGGTTAAGAAGCTGCT	C. maquilingensis fw	PCR, 1st
P-1646	CAAGACCTACGTTTGTGTGATGCAACTTC	C. lagnuensis fw	PCR
P-1647	GCGTACGTCTGCGTCATGCAGCTT	A. aceticus fw	PCR
P-1648	GCAGCTGCACAGGCCCGTGGAGGA	A. camini fw	PCR, 2nd
P-1649	GGTTATACAGCTCCACGGCGATGT	D. mobilis fw	PCR
P-1650	CGTAATGGTAATACAATTCCACGATGCCTT	I. islandicus fw	PCR, 2nd
P-1651	CGTGATGGTGATCCAGCTTCATACA	S. marinus fw	PCR, 2nd
P-1652	AAGGAGTACGTGTGCGTGATGCAG	S. hygrogenophila fw	PCR, 2nd
P-1653	AGAATACGTAATGGTTGTTCAACTACACGA	S. zilligii fw	PCR
P-1654	ATACACAGCGATAAGGAGTACGTG	T. maritiimus fw	PCR, 2nd
P-1655	ATGGTGATACAGTTCCACGGGGAT	T. aggregans fw	PCR
P-1656	TGTGTTATGCAGCTCCACCGTCCA	H. butylicus fw	PCR
P-1657	TGTGCGTAATGCAGCTCCACCGCC	P. occultum fw	PCR, 2nd
P-1658	GATGCAGCTGCACGAGCCAGTAGA	P. fumarii fw	PCR, 2nd
P-1659	TCTGCGTAATGGAAACTCACGAGA	A. infernus fw	PCR
P-1660	ATCTGTCTCATGGAAGTGCATTGC	M. hakonensis fw	PCR, 2nd
P-1662	GGTAAGGAATATGTCTGCATTATGCAAGTG	S. azoricus fw	PCR, 2nd
P-1663	TTTGCTTATTGCAAATGCACTGTAATGTAG	S. acidocaldarius fw	PCR
P-1664	CTGGGAAAGAATATGTATGCTTAATGCAAG	S. ohwakuensis fw	PCR
P-1665	GTGGGTGTTATGCTCCTTCACTCT	C. maquilingensis fw	PCR, 2nd
P-1666	GGTCTATGTGGCTGTGGCTAAGTT	P. oguniense fw	PCR, 2nd
P-1667	TACATAGCCGTCATGACCCTTCAC	T. moditius fw	PCR
P-1668	TATGTCGCCGTCGCCAAGTTTCAC	T. tenax fw	PCR
P-1669	GAAACTTCATGGCGATGTCGATGA	V. distributa fw	PCR, 2nd
P-1676	CCGGCGTTCTACCGGTTGCGTTCA	I. islandicus fw	PCR. 1st
P-1677	CAAAGTGACCGGCGTATTACCTGT	S. marinus fw	PCR. 1st
P-1678	GCGACGAAGATCATCGGAATGGTT	P. fumarii fw	PCR. 1st
P-1679	CCCAAAGTAACGGGAGTATTGCCA	S. azoricus . fw	PCR. 1st
P-1680	TGCCAATTGCAGTGGCTGAGGGAA	P. oguniense fw	PCR. 1st
P-1681	GGCCATGCCCGCGATTAACACACT	V. distributa fw	PCR. 1st
P-1684	CATAGGTTTAGGGAAGGCCACCAA	M. sedura fw	PCR
P-1696	AGCCCTGCAGAGGATGACAAGGAT	T. maritimus fw	PCR. 1st
P-1710	TGCCCGTGGCGCTCCACCGCATGA	S. hygrogenophila fw	PCR. 1st
P-1711	AGGTCTAGGTAAGGCCACAAGAGT	M. hakonensis fw	PCR. 1st
P-1719	CTTCTAACAATAGAATCTGAAGCAGGTACT	C. lagnuensis	sequencing
P-1720	TAAATAATAGTCTTCATTAAAAGGTCCTGT	C. lagnuensis	sequencing
P-1721	TGCCGTCATAAGGTGGCGGGAGGA	A. camini	sequencing
P-1722	TCAGGGAGGAGGGTAAGGATGACT	A. aceticus	sequencing
P-1723	CGACATCGGGCTATTGCTCGGAGT	D. mobilis	sequencing
P-1724	TTGGGCTTGCTAATGGGCATTGGA	I. islandicus	sequencing
P-1725	AGGGACCGCTTCTAGTTCTCCTCA	I. islandicus	sequencing
P-1726	GGGCCCACATGAGGGAGCTGAGGA	T. maritiimus	sequencing
P-1727	CGACGTCCCAGCAGATCTTCCTCA	T. maritiimus	sequencing
P-1728	TCATACTCGGCGTTGGAGCACACA	S. zilligii	sequencing
P-1729	CAGGGTGGACGGGAAGGACGACCT	S. hygrogenophila	sequencing
P-1730	GAATTGAGGAGAACAAGGTCAGGA	M. sedura	sequencing
P-1737	CGATGTCGTGGGCCAGCTTCCTCA	D. mobilis	sequencing
P-1738	TTAGAAGAACACGTACGGGACCTT	S. marinus	sequencing
P-1739	GTACCTGCTTCACATCCTACACGA	S. marinus	sequencing
P-1740	GTAAGTCCCCGCCTCGCAGGCGAT	S. hygrogenophila	sequencing
P-1741	AGTATGAGGCCAATATCGTGTGCT	S. zilligii	sequencing
P-1742	TATACACTGGCGTGGGGGCTCACA	T. aggregans	sequencing

P-1743	CCCAATAGAGTATGCAGTTAGCCA	H. butylicus	sequencing
P-1744	GAGAAAAATATGCCACGATATGGGAA	A. infernus	sequencing
P-1745	TGTAGGGAGGAGGATGAACTGAGA	M. hakonensis	sequencing
P-1746	TGCAAAGATGAGAGTGACCTAAGA	S. azoricus	sequencing
P-1747	TAATATGGTTACACTCCAAGAAGTTTCAGA	S. acidocaldarius	sequencing
P-1748	GAGATTTCAGAAGCTCTTTATATGTGGA	S. ohwakuensis	sequencing
P-1749	GCTTAGGAGGTTAATTAGGCCTGT	C. maquilingensis	sequencing
P-1750	ATCTAAGGAGCGTCTTGTTGCCTA	P. oguniense	sequencing
P-1751	TATCTCTGGAAGCAGTACGGCGAT	T. modiscus	sequencing
P-1752	TATGGAGGAAGTACGGCGACGACA	T. neurotrophilus	sequencing
P-1753	TGGAGGAACTACGGCGATGACACT	T. tenax	sequencing
P-1754	ATTATGGAGTTGAGGACCTGCTCA	V. distributa	sequencing
P-1757	CTGGAGGAAGCAACCAAGGTCATA	P. occultum fw	PCR, 1st
P-1794	CCCGTAGGATTAGATAATGCCACT	S. metallicus fw	PCR, 1st
P-1795	TTGCGTGATGCAGGTTCACTCTGA	S. metallicus fw	PCR, 2nd
P-1796	ATAGCTTATGGTGCAAAGTTAACAGC	S. metallicus	sequencing
P-1797	AAGTTGCTATCTCCATGGGCATGA	S. metallicus	sequencing
P-1816	TCCCTGTTGCCTTAGCGGAAGCTA	I. pacificus fw	PCR, 1st
P-1817	TACGTCATGCTTTCCGGAAAGGAA	I. pacificus fw	PCR, 2nd
P-1861	GGACAGGAACTGCTCTACGATGCA	Thermofilum	sequencing
P-1951	GGGCTATCCCAAAGTAACCGGAGT	A. brierlegi fw	PCR, 1st
P-1952	CATATGTGTAATGCAAGTTCATTGTGA	A. brierlegi fw	PCR, 2nd
P-1953	GCTCGCCAACAGCACCAAGGTCAT	D. mucosus fw	PCR, 1st
P-1954	ATGGTTATACAGCTCCACGGCGAT	D. mucosus fw	PCR, 2nd
P-1955	GTACGAAGGTCATTGGGAACGTGA	D. amylolyticus fw	PCR, 1st
P-1956	ACAGTTACATGGGGATGCCAGGGA	D. amylolyticus fw	PCR, 2nd
P-1957	AGGCCCTGCTTGCTTTATCGCGTT	P. islandicum fw	PCR, 1st
P-1958	CGGCGATGTAGATGAGGAGAAGCT	P. islandicum fw	PCR, 2nd
P-1959	AGGCCCTGCTTGCTTTATCGCGTT	P. organotrophum fw	PCR, 1st
P-1960	TCCACGGCGATGTAGATGAGGAGA	P. organotrophum fw	PCR, 2nd
P-1961	GGTAACCCCAAAGTATCTGGAGTA	A. ambivalens fw	PCR, 1st
P-1962	TCTGCGTAATGGAAACTCACGAGA	A. ambivalens fw	PCR, 2nd
P-1963	AGACCCGAAGGTGACTGGCGTACT	P. abyssi fw	PCR, 1st
P-1964	ACGTGTGTAATGCAGCTCCATCAT	P. abyssi fw	PCR, 2nd
P-1965	TTAAACCCCAAAGTGACCGGCGTA	S. hellenicus fw	PCR, 1st
P-1966	GATCCAGCTTCACGCACCAGTAGA	S. hellenicus fw	PCR, 2nd
P-1967	CGATAGCGCTTGGAGAATCAACGA	V. souniana fw	PCR, 1st
P-1968	CACGGTGATGTAGATGATGGCAAG	V. souniana fw	PCR, 2nd
P-1969	CCAATTGCAGTGGCTGAGGGGACT	P. arsenaticum fw	PCR, 1st
P-1970	GGCTAAGTTCCACGGAGATGTGGA	P. arsenaticum fw	PCR, 2nd
P-1971	GTAACGGGTGTCCTGCCCATAGCA	C. noboribetus fw	PCR, 1st
P-1972	CGTCATGCAGCTTCACGGCGATGT	C. noboribetus fw	PCR, 2nd
P-1973	AGCCCTGGAGGAAGCAACCAAGGT	P. brockii fw	PCR, 1st
P-1974	TGGTGGTGCACACGAGCAAGGAGT	P. brockii fw	PCR, 2nd
P-1975	GTAACTGGAGTATTGCCTGTAGCA	I. aggreganse fw	PCR, 1st
P-1976	GGAGTATGTAGGGGTTATGGAGCT	I. aggreganse fw	PCR, 2nd
P-1985	GTTACGCTTCAAGAGGTCTCGGAA	A. brierlegi	sequencing
P-1986	AACATCTATCGCCCACCTCCCGAA	D. mucosus	sequencing
P-1987	GGAAGAAATACGGCGACGACACGT	P. arsenaticum	sequencing
p-1988	GGCCGTGTACCGCTTCAGGGAGGA	C. noboribetus	sequencing
P-1997	ATTGAGGAGGATAGGTGTGGCACA	I. aggregans	sequencing

*; fw, forward
