# Supplementary material for: Gain and loss of an intron in a protein-coding gene in Archaea: the case of an archaeal RNA pseudouridine synthase gene
Source: BMC Evol Biol. 2009 Aug 11;9:198. doi: 10.1186/1471-2148-9-198 (PMC2738675; doi:10.1186/1471-2148-9-198)
Supplement: Additional file 3 — Alignment of archaeal Cbf5 sequences used in the analysis for Additional file 4. #; selected positions for the analysis. [file 1471-2148-9-198-S3.pdf]

Gblocks 0.91b Results

Number of sequences: 56
Alignment assumed to be: Protein
New number of positions: 201

```
                         10        20        30        40        50        60
                 =========+=========+=========+=========+=========+=========+
P_arsenaticum    ------------------------------------------------------------
P_oguniense      ------------------------------------------------------------
P_aerophilum     -----------------------MKCPSREVFSKFEESTNPQWGKPPSQRSTEEYIKYSL
P_islandicum     ------------------------------------------------------------
T_neutrophilus   ------------------------------------------------------------
P_calidifontis   -----------------------MKCGSREVFVKLEEGTNPQWGKPPSSRSAEEHIRYSF
T_tenax          ------------------------------------------------------------
V_souniana       ------------------------------------------------------------
V_distributa     ------------------------------------------------------------
T_modestius      ------------------------------------------------------------
C_maquilingensi  ------------------------------------------------------------
K_cryptofilum    ------------------------MIDEVEEIAVKSAYEPGPYGYRPQERPIFLYLDHGL
N_equitans       MLCLSLMRIFCSKCLEEQECPLPWELEKYEIWVKKEAETNEKWGEDPYNRPIERLLKYSV
M_kandleri       ----------MSGDKDRRLP----FDRDREMITKAEVETDPRYGCPPEERPIEEYIMKGV
M_janaschii      -------MILLEKTQEKKIN------DKEELIVKEEVETNWDYGCNPYERKIEDLIKYGV
Thermofilum      ------------------------------------------------------------
S_hellenicus     ------------------------------------------------------------
S_marinus        ------------------------------------------------------------
D_mucosus        ------------------------------------------------------------
D_mobilis        ------------------------------------------------------------
D_amylolyticus   ------------------------------------------------------------
S_zilligii       ------------------------------------------------------------
T_aggregans      ------------------------------------------------------------
I_pacificus      ------------------------------------------------------------
I_islandicus     ------------------------------------------------------------
I_hospitalis     ----------VKEALEWYEEMHRKCDFRGKWVIREEAETDPKYGKEPWKRDVKELVELGV
C_noboribetus    ------------------------------------------------------------
A_aceticus       ------------------------------------------------------------
C_lagunuensis    ------------------------------------------------------------
A_pernix         --MAEEAVDARGEGARFIEKVKSICGNTSRILYKYDEPTDPRYGYLPHERPLDVYLRYGM
A_camini         ------------------------------------------------------------
S_hydrogenophil  ------------------------------------------------------------
T_maritimus      ------------------------------------------------------------
P_brockii        ------------------------------------------------------------
P_occultum       ------------------------------------------------------------
P_abyssi         ------------------------------------------------------------
H_butylicus      ------------------------------------------------------------
P_fumarii        ------------------------------------------------------------
A_ambivalens     ------------------------------------------------------------
A_infernus       ------------------------------------------------------------
A_brierleyi      ------------------------------------------------------------
S_tokodaii       -----------MEIYDFIYKIDSFCNYSNNWNIIQDSYTNEKYGYFADKRPIEVLIKNSI
S_ohwakuensis    ------------------------------------------------------------
S_acidocaldariu  ------------------------------------------------------------
S_azoricus       ------------------------------------------------------------
S_shibatae       ------------------------------------------------------------
S_solfataricus   -----------MILNDFIYKIDNFCGYKNEWKIRKDSETSDKYGYYPEKRPIEIHIKNSI
M_haknonensis    ------------------------------------------------------------
M_sedula         ------------------------------------------------------------
S_metallicus     ------------------------------------------------------------
I_aggregans      ------------------------------------------------------------
APKG5E24         ----------------------MTLKQLEGLITIDEDITDNQYGTYYNKRTVEQLLNYGL
APKG8G2          ----------------------MTLKQLEGLITIDEDITDNQYGTYYNKRTVEQLLNYGL
N_maritimus      ----------------------MTLKQLENLIEVDQDITDDAYGTYYDKRTIEQLLNYGI
APKG8O8          ----------------------MKLKQLENLVTVDEDITDDKFGTYYDKRSVEQLLNYGL
C_symbiosum      ----------------------LTLAQLSGLRVIDQDVTDDAHGTYHDKRSVEQLLEYGI
```

```
                          70        80        90       100       110       120
                 =========+=========+=========+=========+=========+=========+
P_arsenaticum    -------------VAAWVKKILG-VERAGHAGTLDPKVSGVLPIAVAEGTKVLMALSRSD
P_oguniense      --------------AAWVKKILG-VERAGHAGTLDPKVSGVLPIAVAEGTKVLMALSRSD
P_aerophilum     VILDKPRGPSSHEVAAWVKKILG-VERAGHAGTLDPKVSGVLPIAIAEGTKVLMALSRSD
P_islandicum     -------------VAAWVKKILG-VERAGHAGTLDPKVSGVLPIAVAEGTKALLALSRSD
T_neutrophilus   --------------AAWVKKILG-VERAGHAGTLDPKVSGVLPIAVAEGTKVLLALSRSD
P_calidifontis   LILDKPRGPTSHEVAAWVKKILG-VERAGHSGTLDPKVSGVLPVAVAEGTKVLMALSRAD
T_tenax          --------------VAWLKRILN-VEVAGHSGTLDPNVSGVLPVAIAEGTKALMALSRAD
V_souniana       --------------AAWIKKMLN-LDRAGHSGTLDPRVSGVLPIALGESTKAMPAINNLD
V_distributa     --------------AAWVKKMLN-LDRAGHSGTLDPRVSGVLPIALGESTKAMPAINTLD
T_modestius      --------------VSWIKKMMG-LERAGHAGTLDPRVSGVLPVALGDSTKVLQGISHED
C_maquilingensi  --------------VAWVKKLLN-ISKAGHSGTLDPGVSGVLPIALGDSTKVLQGISNVD
K_cryptofilum    IILDKPKGPSSHEVTERVKRILEYPGKVGHCGTLDPKVSGVLPIVLGNATKLSKFISGYD
N_equitans       INLDKPSGPTSHQVVAWVRDIVG-V-KAGHGGTLDPKVTGVLPIAIGEATKVLQTLLIAG
M_kandleri       INLDKPAGPTSHEVVAWVKEIFG-LSKAGHGGTLDPKVTGVLPIALEKATKIIQTLLPAG
M_janaschii      VVVDKPRGPTSHEVSTWVKKILN-LDKAGHGGTLDPKVTGVLPVALERATKTIPMWHIPP
Thermofilum      -------------VVAWLRKLLG-IERIAHAGTLDPKVSGVLPITLNNAVRVLPVLLKED
S_hellenicus     --------------VAWIKKMFE-LERAGHGGTLDPKVTGVLPVALANATKVIGNVIHTI
S_marinus        --------------VAWIKKMFE-LERAGHGGTLDPKVTGVLPVALANATKVIGNVIHTI
D_mucosus        --------------VAWVKKMFG-VEKAGHGGTLDPKVTGVLPVGLANSTKVIGNVIHSV
D_mobilis        --------------VAWVKKMFG-VEKAGHGGTLDPKVTGVLPVGLANSTKVIGNVIHSV
D_amylolyticus   --------------VAWVKKMFN-LEKAGHGGTLDPKVTGVLPIGLANSTKVIGNVIHSV
S_zilligii       --------------VAWIKRMFN-VEKAGHGGTLDPKVTGVLPVGLASSTKVIGNVIHSI
T_aggregans      --------------VAWVKKLFN-IDKAGHGGTLDPKVTGVLPVGLDNSTKVIGNVVHSV
I_pacificus      --------------VAWIKKMFN-LEKAGHGGTLDPKVTGVLPVALAEATKVIHYVMLSG
I_islandicus     --------------VAWIKRMFN-LDKAGHGGTLDPKVTGVLPVAFKEATKVIHYVMLSG
I_hospitalis     VNLDKPPGPTSHEVVAWIKRMFG-LERAGHGGTLDPKVTGVLPVALAEATKVIHYVMLSG
C_noboribetus    --------------TAWVKRLLG-VKRAGHGGTLDPKVTGVLPIALEHMTKIMGSVVHSR
A_aceticus       --------------TAWVKRLLG-VKRAGHGGTLDPKVTGVLPVALDHMTKIMGSVVHSR
C_lagunuensis    --------------VAWIKRLLN-VDKAGHAGTLDPRVSGVLPVTLGHMTKIIGYITHSN
A_pernix         IVVDKPPGPTSHEVVAWIKRMLG-VSRAGHGGTLDPKVTGVLPVALERMTRIIGTVMHSS
A_camini         --------------VAWIKRMLG-VSRAGHGGTLDPKVTGVLPVALERMTRIIGTVMHSS
S_hydrogenophil  --------------VAWVKRMFN-VPRAGHGGTLDPRVTGVLPVALHRMTRVVGSVVHSS
T_maritimus      --------------VAWIKRMFN-IKRAGHGGTLDPKVTGVLPVALQRMTRIVGSVIHSD
P_brockii        --------------VAWVKRMFG-LSKAGHGGTLDPKVTGVLPVALEEATKVIGLVVHTS
P_occultum       --------------VAWVKRMFG-LSKAGHGGTLDPKVTGVLPVALEEATKVIGLVVHTS
P_abyssi         --------------VAWVKRMFG-LSKAGHGGTLDPKVTGVLPVALEEATKVIGLVVHTG
H_butylicus      --------------VAWIKRMFG-LSKAGHGGTLDPKVTGVLPVALEEGTKVIGLVVHTG
P_fumarii        --------------VAWIKRMFG-LSKAGHGGTLDPKVTGVLPVALEEATKIIGMVVHTP
A_ambivalens     --------------AFWIKKMFN-LSKVGHGGTLDPKVSGVLPIGLENATKLMTYITKAG
A_infernus       --------------AFWIKKMFN-LNKVGHGGTLDPKVSGVLPIGLENATKLMTYITKAG
A_brierleyi      --------------AFWVKKMFN-INKVGHGGTLDPKVTGVLPIGLENATKIMNYVTEAG
S_tokodaii       INADKPPGPTSHEVAYWIKQMFK-VSKAGHGGTLDPKVTGVLPIGLENATKLMSYISSSG
S_ohwakuensis    --------------AYWIKQMFK-VSKAGHGGTLDPKVTGVLPIGLENATKLMSYISSSG
S_acidocaldariu  --------------AYWVKKLFN-ITKAGHGGTLDPKVTGVLPIGLENATKLMNYISKSG
S_azoricus       --------------AYWVKKMFN-LNKVGHGGTLDPKVTGVLPIGMENATKLMSLISKAG
S_shibatae       --------------AYWVKKMLN-VSKAGHGGTLDPKVTGVLPIGIENATKIMNYISKGG
S_solfataricus   INLDKPPGPTSHEVAYWVKKMLN-VTKAGHGGTLDPKVTGVLPIGIENATKIMNYISKGG
M_haknonensis    --------------AFWVKTMFG-LPRVGHGGTLDPKVTGVLPIGLGKATRVMNLITKSG
M_sedula         --------------AFWVKTMFN-LPRVGHGGTLDPKVTGVLPIGLGKATKIMSVVTKSG
S_metallicus     --------------AFWIKQRLS-LNRVGHGGTLDPKVTGVLPVGLDNATKIMHYVSRST
I_aggregans      --------------VAWIKKMFN-IPKAGHGGTLDPKVTGVLPVALDRATRVIGILMHSY
APKG5E24         ILLDKPAGPTSHEVVAWTKRILE-IPKAGHSGTLDPQVTGVLPLGLGEGTKALGVLLLGP
APKG8G2          ILLDKPAGPTSHEVVAWTKRILE-IPKAGHSGTLDPQVTGVLPLGLGEGTKALGVLLLGP
N_maritimus      ILLDKPPGPTSHETVAWTKRILK-LPKIGHSGTLDPQVSGVLPLGLGEATKALGVLLFGP
APKG8O8          ILLDKPPGPTSHQVVAWAKKILE-IPKAGHSGTLDPQVTGVLPLGLGDATKALGVLLFGS
C_symbiosum      VLLDKPPGPTSHEAVAWAKRILE-IPKAGHSGTLDPQVSGVLPLGLGEATKALGVLLIGP
                                 #####     ##################################
```

```
                          130       140       150       160       170       180
                  =========+=========+=========+=========+=========+=========+
P_arsenaticum    KVYVAVAKFHGDVDEERLRAVLREFQGEIYQKPPLRSAVKRQLRTRRVFSLELLELEG--
P_oguniense      KVYVAVAKFHGDVDEERLRAVLREFQGEIYQKPPLRSAVKRQLRTRRVFSLELLELEG--
P_aerophilum     KVYVAVAKFHGDVDEDKLRAVLQEFQGVIYQKPPLRSAVKRQLRTRRVYSLDLLELDG--
P_islandicum     KVYVAVAKFHGDVDEEKLKAVLNEFQGVIYQKPPLRSSVKRQLRTRHVYSLELLELDG--
T_neutrophilus   KVYVAVAKFHGDVDEGKLRAVLGEFQGIIYQRPPLRSAVKRQLRTRQVYSIELLELDG--
P_calidifontis   KVYIAVAKFHGDVDVENLRRVLQELQGEIYQKPPLRSAVKRQLRTRRVYSLELLELDG--
T_tenax          KTYVAVAKFHGDVDEAKLREVLAYFTGAIYQRPPLRSAVKRQLRVRHVYSLELLELDG--
V_souniana       KEYIMVMKLHGDVDDGKLRAVLREFTGAIYQRPPLRSAVKRQLRVKHVYELELLERDG--
V_distributa     KEYIMVMKLHGDVDDEKLRAVLREFTGAIYQRPPLRSAVKRQLRVKHVYELELLERDG--
T_modestius      KEYIAVMTLHGDASDERIREVLAEFTGEIYQRPPVKSAVKRQLRTRRVYSLKMLERDG--
C_maquilingensi  KEYVGVMLLHSTVDQERVKAVFREFTGKIYQRPPLKSAVKRRIRVKTVYSLDIMEFDG--
K_cryptofilum    KEYVGTLYLHGDVPIDELKGALDKFTGPIFQRPPVKSAVKRSLRVRRVYSIELLSSEG--
N_equitans       KEYVALMHLHKEVSEKDIIKVMSKFVGTIIQTPPLRSAVKKRPRKKKVYCIKIIEIDG--
M_kandleri       KEYVTIMHLHGDVDEEELERVVKEFEGTILQRPPLRSAVKRRVRPKKIYYIDILEIDG--
M_janaschii      KEYVCLMHLHRDASEEDILRVFKEFTGRIYQRPPLKAAVKRRLRIRKIHELELLDKDG--
Thermofilum      KEYVCVMRLHGDVDPERLERVVSMFKGRIYQRPPLRSAVKREVRIRQIYDIRLLEFNE--
S_hellenicus     KEYVMVIQLHAPVDNDRLRKVLKYFTGVIYQRPPLRSSVKRVIRTRRIHYIDLLEHS--D
S_marinus        KEYVMVIQLHTPVDNERLREVLRYFTGVIYQRPPLRSSVKRVIRTRRIHYIDLLEHS--D
D_mucosus        KEYVMVIQLHGDVAEQDLRRVAGYFRGEIYQRPPLRSSVKRAIRVRRIHEIEVLEVR--G
D_mobilis        KEYVMVIQLHGDVAEQDLRRVAGYFRGEIYQRPPLRSSVKRAIRVRRIHEIEVLEVR--G
D_amylolyticus   KEYVMVIQLHGDARESSVKMVVEYFKGDIYQKPPLRSSVKRSIRVRRIYEIELLEIR--D
S_zilligii       KEYVMVVQLHDLVERKKVEEVVEYFKGQIYQRPPLRSSVKRSIRVKTIYEIEILDVQ--D
T_aggregans      KEYVMVIQFHGDVEIGKLVEALKYFTGEIYQRPPLRSSVKRVLRTRRIHEIELLDFK--D
I_pacificus      KEYVMVIQFHDAVKEQDVIENLKYLVGEIYQRPPLRSSVKRQLRTKKVYYIHLIEFYPER
I_islandicus     KEYVMVIQFHDAFNEKDVIENLKYLVGEIYQRPPLRSSVKRQLRTKKVYYIHLIEFYPER
I_hospitalis     KEYVMVIQFHDAVKEEEVIENLKYLVGEIYQRPPLRSSVKRQLRTKKVYYIHVLEFWPER
C_noboribetus    KAYVCVMQLHGDVSEERLRDVAKEFTGTIYQRPPVRSNVKRALRTRRIFSLEVLEVRG--
A_aceticus       KAYVCVMQLHGDVSEKKLREVAEEFTGTIYQRPPVRSNVKRALRTRRIFSLEVLEVRG--
C_lagunuensis    KTYVCVMQLHKDVDQNELKRVINDFIGTIYQKPPIRSHVKRTLRTRKIFNITINEIVD--
A_pernix         KEYVCVMQLHRPVEEDRLREVLKLFEGEIYQKPPLRSSVKRALRTRRVFRIELLEYTG--
A_camini         KEYVCVMQLHRPVEEGRLREVLRLFEGEIYQKPPLRSSVKRALRTRRIFRIELLEYTG--
S_hydrogenophil  KEYVCVMQLHRPVEEERLREALRLFEGRIYQRPPLRSSVKRSLRVKRVDEIELLEYDG--
T_maritimus      KEYVCVMQLHAPVEEERLREVLNEFEGVIYQRPPLRSSVKRALRKKRVYKIELLEYTG--
P_brockii        KEYMCVMQLHRPVPEEELRRVLGMFVGEIYQRPPLRSSVKRSLRVKRIYEIELLEYNG--
P_occultum       KEYMCVMQLHRPVPEEELRRVLGMFVGEIYQRPPLRSSVKRSLRVKRIYEIELLEYNG--
P_abyssi         KEYTCVMQLHHPVPEQDLRRALEMFVGEIYQRPPLRSSVKRSLRTKKIYEIELLEYNG--
H_butylicus      KEYMCVMQLHRPVPEEELRRAINMFVGEIYQRPPLRSSVKRSLRLKKIYEIELLEYNG--
P_fumarii        KEYICVMQLHEPVEEKKLLEAIKVFTSTIYQRPPLRSSVKRSLRTKTIYEIELLEYTG--
A_ambivalens     KEYICVMETHEKVDLSIVKKIAEEFKGTIYQRPPVRSSVKRRLRTRKVFDIEVLEGKDD-
A_infernus       KEYICVMETHEKVDLSIVKKIAEEFKGTIYQRPPVRSSVKRRLRTRKVFDIEVLEGKDD-
A_brierleyi      KEYICVMQVHCDIEKDLLKSIIEKFKGKIYQRPPVRSSVKRRLRFRTVNEIELLETYN--
S_tokodaii       KEYVCLMQVHCDFNIDELKQIISKFIGIIYQKPPVRSSVKRRTRKKKIYDIEILDTDK--
S_ohwakuensis    KEYVCLMQVHCDFNIDELKQLISKFIGIIYQKPPVRSSVKRRTRKKKIYDIEILDTDK--
S_acidocaldariu  KEYICLLQMHCNVDQKELKEIISQFVGEIYQKPPVRSSVKRRIRKRRIYAIDILDMQD--
S_azoricus       KEYVCIMQVHCDYKMDELKEIVNQFIGEIYQRPPVRSSVKRRTRKRRVYSIDILETEG--
S_shibatae       KEYVCVMQVHCEYDKEELTKIIKSFEGEIYQRPPVRSSVKRRLRVRSIYDIEILDMDK--
S_solfataricus   KEYVCVMQVHCEYNKEELAKIISSFKGEIYQRPPVRSSVKRRLRIRRIYDIEILDMDK--
M_haknonensis    KEYICLMEVHCEFSEDKLREVSKQFVGTIYQKPPVRSSVKRRVRKREIYSLDLLEISG--
M_sedula         KEYVCLMEVHCDFQEERLRAIAKEFVGTIYQKPPVRSSVKRRVRKRKIYSLEVLEMEG--
S_metallicus     KEYVCVMQVHSDFEKESLKSWIMKFKGKIYQRPPVRSSVSRKLRTRKIYDIEVTEIED--
I_aggregans      KEYVGVMELHGDVEPEKVIETMKMFVGKIYQRPPLRSSVKRSLRIREIYSIDVLEIEN--
APKG5E24         KEYHALGRLHSLPSKEKLENILNEFCGDIYQKPPQRSSVLRQTRVRKIFELELLEQKE--
APKG8G2          KEYHALGRLHSLPSKEKLENILNEFCGDIYQKPPQRSSVLRQTRVRKIFELELLEQKE--
N_maritimus      KEYHALGRVHSLPSKEKLHEVIESLTGEIYQKPPQRSAVVRQTRTRTIYEFEVLEQKE--
APKG8O8          KEYQGLGRLHLLPSKEKLDGILKEFCGDIYQRPPQRSAVSRQTRIRKIFELELLEQKE--
C_symbiosum      KEYVAVGRFHALQDSEKLGELARLFTGPIHQKPPQRSSVLRRTRVKTIHEIEVLEQKE--
                 #######################################################
```

```
                         190       200       210       220       230       240
                 =========+=========+=========+=========+=========+=========+
P_arsenaticum    -RYAVIKMHVEAGTYARKIIHDIGEVLGVGANMRELRRVAVTCFTED-E-AVTLQDVADA
P_oguniense      -RYAVIKMHVEAGTYARKIIHDIGEVLGVGANMRELRRVAVTCFTED-E-AVTLQDLADA
P_aerophilum     -RYAVIKMHVEAGTYARKIIHDIGEVLGVGANMRELRRIAVSCYTED-E-AVTLQDIADA
P_islandicum     -RYAVIKMHVEAGTYARKLIHDIGEVLGVGANMRELRRIAVSCYTED-E-TVALQDVADA
T_neutrophilus   -RYAVIKMHVEAGTYARKLIHDIGEVLGVGANMRELRRVAVSCYTEE-E-TVTLQDVADA
P_calidifontis   -RYAVLKMHVEAGTYARKLIHDLGEILGVGANMRELRRVAVSCFTED-E-AVTLQDLADA
T_tenax          -RYALLRMHVEAGTYARKLIHDIGEVLGVSANMRELRRVGVGCFTEE-E-AFTLQDIADA
V_souniana       -KYALIRMNVESGTYARKLAYDIGEVLGVGANMRELRRIRVGCFTEK-E-AITLQDLKDA
V_distributa     -KYVLIRMNVESGTYARKLAYDIGEVLGIGANMRELRRTRVGCFTEK-E-AVTLQDLKDA
T_modestius      -RYVLLDARVEAGTYIRKLCYDVGEVLGVGANMRELRRVRVGCFGED-E-AVTLHDLADA
C_maquilingensi  -RYVLFKADVESGTYIRKLCYDIGEVLGVGASMRELRRTRVGCFREE-N-TVNLNTLREA
K_cryptofilum    -RFHKLRVRVESGTYIRKLFFDIGEFLGVGGSMRDLRRIRSGIFTEK-D-CVTLEDIKDA
N_equitans       -KDVLFRVSTQGGVYIRKLIHDIGVKLGVGAHMQELRRIKSGPFHEN-N-SVYLQDIVDS
M_kandleri       -RDVLMRVGCQAGTYIRKLCHDIGEALGVGAHMAELRRTRTGPFSEE-N-AVTLHDVKDA
M_janaschii      -KDVLFRVKCQSGTYIRKLCEDIGEALGTSAHMQELRRTKSGCFEEK-D-AVYLQDLLDA
Thermofilum      -RTALLHVWCEAGTYMRKLCHDIGEILGVGAHMQELRRIRSGSLYEDRN-CSTMHDVVDA
S_hellenicus     -RYVLVRVGCEAGTYMRKLAHDIGLLLGVGAHMRELRRTRTGPYKEDET-LVRMQEVSEA
S_marinus        -RYVLVRVGCEAGTYMRKLAHDIGLLLGVGAHMRELRRTRTGPYKEDET-LVRMQEVSEA
D_mucosus        -RFALIRVLSDPGTYMRKLAHDIGLLLGVGAHMRELRRTRTGPYREDET-LVRLQDVSEA
D_mobilis        -RFALIRVLSDPGTYMRKLAHDIGLLLGVGAHMRELRRTRTGPYREDET-LVRLQDVSEA
D_amylolyticus   -RFILVRVLSDPGTYMRKLAHDIGLMLGTGAHMRELRRTRTGPYREDET-LVRLQDISEA
S_zilligii       -KFLLLRVLCDPGTYMRKLAHDIGLILGVGAHMRELRRTRTGPFREDET-LVSLQEVSEA
T_aggregans      -RFALVRASCDPGTYMRKLAHDIGLYTGVGAHMRELRRTRTGPYREDET-LVKLQDVSEA
I_pacificus      -RMALVRVGSESGTYMRKIAHDLGLLLGTGAHMRELRRTRSGPFHEEYN-LVRMQELSEA
I_islandicus     -RMVMVRVGSESGTYMRKIAHDLGLLMGIGAHMRELRRTRSGPFHEGYN-LVTMQELSEA
I_hospitalis     -RMALVRVGSESGTYMRKLAHDLGLLVGTGAHMRELRRTRSGPFHEDWN-LVRMQDLSEA
C_noboribetus    -RLALLRVESDPGTYMRKLCWDMGLVLGVGAHMRELRRVKTGPFDEDHN-LVTLQELSEA
A_aceticus       -RLALLRVESDPGTYMRKLCWDMGLVLGVGAHMRELRRIKTGPFDEDHN-LVTLQELSEA
C_lagunuensis    -RKVLLTIESEAGTYMRKLCWDIGLILGIGAHMRELRRIKTGPFNEDYY-LVTMQDLTEA
A_pernix         -KYALLRVDCEAGTYMRKLCWDIGLVLGVGAHMRELRRIRTGPFSEDSGLMVRLDDVAYA
A_camini         -KYALLRVDCEAGTYMRKLCWDIGLVLGVGAHMRELRRVRTGPFSEDSGLMVRLDDVAYA
S_hydrogenophil  ERYALLRIACEAGTYMRKICWDVGLVLGVGAHMRELRRTRTGPFSEARG-LVTLQEVSEA
T_maritimus      -KYALLHIWSEAGTYMRKICWDVGLVLGTGAHMRELRRIRTGPFTEERG-LVRMQDIAYA
P_brockii        -RYALMRVLCEAGTYMRKLCHDAGLILGVGAHMRELRRTKSGPFREQHG-LVRLQELSEA
P_occultum       -RYALMRVLCEAGTYMRKLCHDAGLILGVGAHMRELRRTKSGPFREQHG-LVRLQELSEA
P_abyssi         -RYALMRVFCEAGTYMRKLCHDVGLILGVGAHMRELRRTKSGPFREQHG-LVRLQDLSEA
H_butylicus      -RYALMRVFSEAGTYMRKLCHDIGLILGVGAHMRELRRTRSGPFRERNG-LVRLQELSEA
P_fumarii        -RYALMRVLCDPGTYMRKLCHDIGLYLGVGAHMRELRRTKSGPFREAYG-LVKLQDLSEA
A_ambivalens     -KLFLLRISSEPGTYMRKICHDMGIILGTGAHMRELRRIRSGIFTES-G-LFTLQEVSEA
A_infernus       -KLFLLRISSEPGTYMRKICHDMGIILGIGAHMRELRRIRSGIFTES-G-LVTLQEVSEA
A_brierleyi      -RMALLRISSEPGTYMRKICHDAGILLGCGAHMRELRRIRSGIFTEK-N-LVTLQEVSEA
S_tokodaii       -RFILLRISSDPGTYMRKLCHDIGVILGCGAHMRELRRIRSGIFTEK-N-LVTLQEISEA
S_ohwakuensis    -RFILLRISSDPGTYMRKLCHDIGVILGCGAHMRELRRIRSGIFTEK-N-LVTLQEISEA
S_acidocaldariu  -KLILLRVQSDAGTYMRKLCHDVGVIAGCGSHMRELRRIRSGIFTEKTN-MVTLQEVSES
S_azoricus       -RLVLMKIASEPGTYMRKICHDIGILLGCGAHMRELRRTRSGIFTEDSN-LVTLHEISEA
S_shibatae       -KLVLFRVSCDSGTYMRKLCHDIGIIYGCGAHMRELRRTRSGIFTESTN-LVKLHDLSEA
S_solfataricus   -KLVLLRVNCDSGTYMRKLCHDIGIIYGCGAHMRELRRTRSGIFTESTN-LVKLHELSEA
M_haknonensis    -RRVLMRISSEPGTYMRKICHDMGILLGCGAHMRELRRTKSGIFKED-T-LVTLQQVSEA
M_sedula         -RRVLMKISSEPGTYMRKICHDMGILLGCGAHMRELRRTRSGIFTED-T-VVTLQQVSEA
S_metallicus     -RLVLMKVISEHGTYMRKLCHDIGILSGYGAHMRELRRTRTGIFDER-N-LVTMHEFSEA
I_aggregans      -RRVLFRVKCQSGTYVRKLCHDIGLLLGVEAHMRELRRIGVAHFTENRD-IVTLHEVSEA
APKG5E24         -NLILIRVLCEAGTYIRKLIYDFGEILVHGATMIELRRTRVSQFHENYP-LVTLHQIAVA
APKG8G2          -NLILIRVLCEAGTYIRKLIYDFGEILVHGATMIELRRTRVSQFHENYP-LVTLHQIAVA
N_maritimus      -RLLLTRVLCEAGTYIRKLYYDLGEILGPGATMIELRRTRVDQFRETDG-LVTLHELANA
APKG8O8          -RLILIRITCESGTYIRKLIYDIGEVLGPGATMVELRRTRVNQFHENHP-LVTLHQVADA
C_symbiosum      -RLVLMRVSCEAGTYIRKLLYDMGEVVGSGASMIELRRTRVDHFTEGSG-LVTMHELAEA
                  #############################################    ##########
```

```
                          250       260       270       280       290       300
                 =========+=========+=========+=========+=========+=========+
P_arsenaticum    YYIWKKYGDDTYLRSVLLPIEEIARHLPKIWVRDSAVDAVCHGAPLAAPGISKFEVPFSK
P_oguniense      YYIWKKYGDDTYLRSVLLPIEEIARHLPKIWVRDSAVDAVCHGAPLAAPGISKFEVPFSK
P_aerophilum     YYIWKHYGDDTYLRRVLLPIEEIARHLPKIWVRDSAVDAICNGAPLAAPGISKFETPFSK
P_islandicum     YYIWRRYGDDTYLRRVLLPIEEIARHLPKIWVRDSAVDALCNGAPLAAPGVVKFETPFSK
T_neutrophilus   YYIWRKYGDDTYLRRVLLPIEEIARHLPKIWVRDSAVDALCNGAPLAAPGVAKFEAPFSR
P_calidifontis   YYIWKKYGDDTYLRRVLLPIEEIARPLPKIWVRDSAVDALCNGAPLAAPGVAKFEHPFSR
T_tenax          VYIWRNYGDDTVLRQIVKPIEEIARGVAKIWIKDGAVDAVCHGAPLAAPGVVKFEEPFQR
V_souniana       YTLYRDYGIEDLLRTYVKSVEYMVGHLPKVWVRDSAVDAICHGAALAVPGIVKLTNNIKR
V_distributa     YTLYKDYGVEDLLRTYVKPVEYMVRHLPKVWIRDSAVDAICHGAALAVPGIVKLTNNIGK
T_modestius      LYLWKQYGDESLLRRYVKPVEYMVKHLPRIFIRDSAVDSVAHGAALAAPGVAKIEDGVER
C_maquilingensi  YSLWRDYGDESLLRRLIRPVEEMVIHLPRIYIRDSAVDAIAHGASLAAPGVAKVEEGIRS
K_cryptofilum    YDSWRESGDESKIRKVILPLEEAVRHLPKIYVKDSAVASLTHGASLKVKGICSLSRGIKK
N_equitans       LYFWKEEGNEEYIRKVFLPVEEAVKHLKKIYILDSAVAAIVHGANLAVPGIAKLYSNIKK
M_kandleri       YEFWKEEGWEEPLRHVVRPMEEGLEHLPRIEIRDTAVDAICHGANLAAPGIVRVEKGIQP
M_janaschii      YVFWKEDGDEEELRRVIKPMEYGLRHLKKVVVKDSAVDAICHGADVYVRGIAKLSKGIGK
Thermofilum      YYIWKERGIDHFLRQVFLPVEAAIQHLPKVWIRDSAVDAVCHGAPLAVPGIVKLEGGIKV
S_hellenicus     LYLWRNKGDERYLRKIILPVETAIAHLPKIIIRDTAVDAIAHGAHLAVPGIARLTRDVAP
S_marinus        LYLWRNKGDERYLRKIILPVETAIAHLPKIIIRDTAVDAVAHGAHLAVPGIVRLTKDVAL
D_mucosus        LLLWRSNGDERYLRRIVLPVETSIAHLPKIMILDTAVDAIAHGANLAAPGVARLTRNIAK
D_mobilis        LLLWRSNGDERYLRRIVLPVETSIAHLPKIMILDTAVDAIAHGANLAAPGVARLTRDIAK
D_amylolyticus   LALWRSSGDERYIRRIILPVETSIAHLPKIMILDTAVDAIAHGANLAVPGIARLTSNVER
S_zilligii       LYLWRTRGDERYLKKVVLPVEVSTAHLPKILVLDTAVDAIAHGANLAAPGVSMLTRNVEA
T_aggregans      LYLWRVEKDERMLRRVILPVETAVTHLPKIVINDLAVDAIAHGASLAAPGVVRLTDNVSA
I_pacificus      KFLWDHLKDDSLLKKYIMPCEYAVCHMQKIMVGDGAVDAIAHGAHVAAPGVAALTDGIKK
I_islandicus     KFLWEEKKDDSLLRKYVMPCEYSVCHMQKVMVMDGAVDAIAHGAHVAAPGVAALTDGIRK
I_hospitalis     KFLYDNYGDDSLLKKYIMPCEVATCHMPKIMIKDGAVDAVAHGANVSIRGVAALTDNMKK
C_noboribetus    VYRFREEGKDDYLRRAVLPGEVITCELPKVVLRDSAVESVVNGAPLAAPGVSLLTSDVKA
A_aceticus       VYRFREEGKDDYLRRAVLPGEVITCELPKVVLRDSAVESVVNGAPLAVPGISLLTPDVKA
C_lagunuensis    VYRFKTEEKDDLLRKVIIPGEYSVCELPKVLVRDTAVESVINGSPLAIPGISYYNEGINR
A_pernix         VIRWREEGKDDLLRRVVIPGEYSVCHIPKVLVRDSAVESLTHGAQLAAPGVAAVEEGVEK
A_camini         VIRWREEGKDDLLRRVVIPGEYSVCHIPKVLVRDSAVESLTHGAQLAAPGVAAVEEGVEK
S_hydrogenophil  LYRLRVDGKDDLLRRVVLPGEYSVCHLPKVVVRDTAVESLVHGASLAVPGVVMLHEDIKR
T_maritimus      LIRYRDEGKEDLLRKYVLPGEYSVCHLPKVVVRDSAVESIVNGAMLAVPGIAMLHEGIEK
P_brockii        LYRWKNEGKEDLLRSYIKPVEYAVSHLPKIVIRDTAVDAIAHGANLAVPGIARLHEGIKR
P_occultum       LYRWKNEGKEDLLRSYIKPVEYAVSHLPKIVIRDTAVDAIAHGANLAVPGIARLHEGIKR
P_abyssi         LYRWRNEGKEDLLRKYIKPIEYAVSHLPKIVIRDTAVDAIAHGANLAVPGIVRLHEGIQR
H_butylicus      IYRWKQEGKEDLLRKYILPIEYAVSHLPKIVVRDTAVDAIAHGANLAVPGIARLHEGIKR
P_fumarii        LYRWKQEGKDDLLRKYILPMEYAVAHLKKVVIRDSAVDAIAHGAHLAVPGIARLHADIKK
A_ambivalens     LYMWKNCKDETDLRKILLPMEIGLCGIPKIILDDNAVNAIAYGATANVPGIVAYQN-FKK
A_infernus       LYMWKNCKDETDLRKILLPMEIGLCGIPKIILDDNAVNAIAYGATANAPGIVAYQN-FKK
A_brierleyi      LYMWKSCKDEEDLRKILIPMEMAFCGIPKIVIDDNAVDAIAYGASVMIPGIVAFQN-FKK
S_tokodaii       LYMWKNCKDESDLRKILLPMEYATCGMPKILIDDNAVDAISYGAMLTAPGIVAYQR-FRV
S_ohwakuensis    LYMWKNCKDESDLRKILLPMEYATCGMPKILIDDNAVDAISYGAMLTAPGIVAYQR-FRV
S_acidocaldariu  LYLYRNCKDESELRRILLPMEYGVCGIPKVIVSDTAVNAITYGAKLNLPGILAYQN-FRK
S_azoricus       LYMYKHCKDESDLRRILLPMEFAVCGIPKVVIDDDAVNAITYGATLNAPGVVAFQN-FKK
S_shibatae       IYLYKNCKDETELRRVLMPMEYATCEMPKLIIEDSAVNALAYGAQLAVPGIVAYQN-FKR
S_solfataricus   IYLYKNCKDETELRRVLIPMEFATCEIPKIVIEDSAVNALAYGAQLAVPGVVAYQN-FKK
M_haknonensis    LYLYNNCREEDELRRILIPMEYAFCGIPKIIVDDETVNSLAYGSPLMAPGIVAFQP-FKK
M_sedula         LYLYRNCGEEDELRRILIPMEMAFCGIPKIIVDDETVNSLAYGSPLMAPGIVAFQG-FKK
S_metallicus     VYMWKNCKDESFIRKIIMPMEIATCGIPKIMVDNNAVSAIAYGAKLTAPGVVGFQQ-FKK
I_aggregans      LYIWRNLGDESFLRKMILPVEYIVAFLPKIVVKDSAVDAIAHGAQLAVPGISIVAKNINR
APKG5E24         FADWKDSKDDSKLSTMIHPIEHVLSEIKSVVIRDTAVDALCHGAQLAIPGILQISPNLQK
APKG8G2          FADWKDNKDDSKLSTMIHPIEHVLSEIKSVVIRDTAVDALCHGAQLAIPGILQISPNLQK
N_maritimus      FALWEEKKDDSKLKSMIQPVEHALSELKSVVIRDSAIDAMCHGAQLAIPGILQISPSLNK
APKG8O8          FSDWKEKKDSTKLLKLIHPIENALSELKSIVIRDSAVDALCHGAQLAIPGILQISPNLQK
C_symbiosum      YAVWKEG-DGSRLQRIIRPVEEALAGIKAVVIRDSAVDALCHGAQLAIPGILQVSDNLRI
                 ####    ###########################################        #
```

```
                         310       320       330       340       350       360
                  =========+=========+=========+=========+=========+=========+
P_arsenaticum     GDIVAMF-----------------------------------------------------
P_oguniense       GDIVAMF-----------------------------------------------------
P_aerophilum      GDLVAMFTLKGELIGIGRALVGSEEVKKMERGLVARTDRVVMRRGTYPAMWKRKAKSQSD
P_islandicum      GELVAFF-----------------------------------------------------
T_neutrophilus    GELVAYF-----------------------------------------------------
P_calidifontis    GDLVAYFTLKGELIGIGRALVDSEEVKKMEKGLVARTDRVVMPRGTYPPMWRRGGKSFKS
T_tenax           GDLVAYF-----------------------------------------------------
V_souniana        KSLTAIM-----------------------------------------------------
V_distributa      GSLVAIM-----------------------------------------------------
T_modestius       DRMAAIV-----------------------------------------------------
C_maquilingensi   GQLVALM-----------------------------------------------------
K_cryptofilum     GSIVALMTLKGELIAIGRALMDFDEMLSADSGVAASIERVIMPRDLYPPMWKTG------
N_equitans        GDLVSIHTLKGELVAIGIALMDSKEMLEKKRGIAVDIERVFMKPGLYPKMWVSQG-----
M_kandleri        GDLVAIFTLKGEAVALGVAKATWKEMLHADRGIMVDTKRVLMEPGTYPKAWGLKTPGE--
M_janaschii       GETVLVETLKGEAVAVGKALMNTKEILNADKGVAVDVERVYMDRGTYPRMWKRKK-----
Thermofilum       NSTVAIL-----------------------------------------------------
S_hellenicus      NKTVAIL-----------------------------------------------------
S_marinus         NKTVAIL-----------------------------------------------------
D_mucosus         GSTVAIL-----------------------------------------------------
D_mobilis         GSTVAIL-----------------------------------------------------
D_amylolyticus    NKTVAIL-----------------------------------------------------
S_zilligii        GKTVAIY-----------------------------------------------------
T_aggregans       GATVAVF-----------------------------------------------------
I_pacificus       GDVVAIV-----------------------------------------------------
I_islandicus      GDVVAIF-----------------------------------------------------
I_hospitalis      GDVVAVVSLKGELVAIAQALVSSQEALKMEKGWVAKTKRVIMKPGTYPDVWRKKKASQQE
C_noboribetus     GSQVAMF-----------------------------------------------------
A_aceticus        GGRVAML-----------------------------------------------------
C_lagunuensis     GDLVSLL-----------------------------------------------------
A_pernix          GGMVALMTLKGELIGLGKALASAQEMLEAERGIVVSPTRIIMERGLYPRMWKRQQAPQGA
A_camini          GGMVALM-----------------------------------------------------
S_hydrogenophil   GETVAVL-----------------------------------------------------
T_maritimus       GDTVALL-----------------------------------------------------
P_brockii         GDLVALF-----------------------------------------------------
P_occultum        GDLVSLF-----------------------------------------------------
P_abyssi          GDLVALF-----------------------------------------------------
H_butylicus       GDLVALL-----------------------------------------------------
P_fumarii         GDVVAIF-----------------------------------------------------
A_ambivalens      GDLVGLI-----------------------------------------------------
A_infernus        GDLVGLI-----------------------------------------------------
A_brierleyi       GDLVGI------------------------------------------------------
S_tokodaii        KDTVAILTLKGELVAIGEADVDSQKLVDMKKGIVVKPKRVLMPRDIYPRSWKKHG-----
S_ohwakuensis     KDTVAIL-----------------------------------------------------
S_acidocaldariu   NQDVAVL-----------------------------------------------------
S_azoricus        GENVAIM-----------------------------------------------------
S_shibatae        NDTVAVL-----------------------------------------------------
S_solfataricus    NDTVAVLTLKGELVATGNALMDSEELENAKKGIVVNLSRVFMQRDIYPKAWKKHES----
M_haknonensis     GDVVALI-----------------------------------------------------
M_sedula          GDTVALI-----------------------------------------------------
S_metallicus      DDVVCVITTKGELVSVGKALMDYRRLAKVDKGEVASTDRVFIDRDVYPKHWGDKDGGSS-
I_aggregans       DDRVAIF-----------------------------------------------------
APKG5E24          EDLVGIYTQKGEIVALAQSLMSEDDIKENTKGYAFETKRIIMAPETYPKSWRSRLTIKEN
APKG8G2           EDLVGIYTQKGEIVALAQSLMSEDDIKENTKGYAFETKRIIMAPETYPKSWRSRSTIKEN
N_maritimus       GDIVGIYTQKGEAVALAEATMSGQEIQDAVKGYAFETKRIIMAPNTYPKKWRTKPSSKE-
APKG8O8           DELVGIYTQKGEVVALAQSSMSGETIEETPKGHAFETKRIIMAPNTYPKKWRTRHSKEKT
C_symbiosum       GDLAGVYTQKGEVVALAEAEMEAEVIADATKGHAFKTKRLIMKTDTYPKSWHSKGTYKEK
                  ####
```

```
                 ======
P_arsenaticum    ------
P_oguniense      ------
P_aerophilum     SA----
P_islandicum     ------
T_neutrophilus   ------
P_calidifontis   GT----
T_tenax          ------
V_souniana       ------
V_distributa     ------
T_modestius      ------
C_maquilingensi  ------
K_cryptofilum    ------
N_equitans       ------
M_kandleri       ------
M_janaschii      ------
Thermofilum      ------
S_hellenicus     ------
S_marinus        ------
D_mucosus        ------
D_mobilis        ------
D_amylolyticus   ------
S_zilligii       ------
T_aggregans      ------
I_pacificus      ------
I_islandicus     ------
I_hospitalis     GG----
C_noboribetus    ------
A_aceticus       ------
C_lagunuensis    ------
A_pernix         ------
A_camini         ------
S_hydrogenophil  ------
T_maritimus      ------
P_brockii        ------
P_occultum       ------
P_abyssi         ------
H_butylicus      ------
P_fumarii        ------
A_ambivalens     ------
A_infernus       ------
A_brierleyi      ------
S_tokodaii       ------
S_ohwakuensis    ------
S_acidocaldariu  ------
S_azoricus       ------
S_shibatae       ------
S_solfataricus   ------
M_haknonensis    ------
M_sedula         ------
S_metallicus     ------
I_aggregans      ------
APKG5E24         VTNT--
APKG8G2          VTNT--
N_maritimus      ------
APKG8O8          KTDGGE
C_symbiosum      E-----
```

Parameters used Minimum Number Of Sequences For A Conserved Position: 29 Minimu
m Number Of Sequences For A Flanking Position: 47 Maximum Number Of Contiguous
Nonconserved Positions: 10 Minimum Length Of A Block: 5 Allowed Gap Positions:
None Use Similarity Matrices: Yes
Flank positions of the 6 selected block(s) Flanks: [77 81] [87 175] [182 2

26] [231 244] [249 291] [300 304]   New number of positions in /Users/yokobori/Desktop/Cbf5_2/list080725.fasta-gb: 201 (54% of the original 366 positions)
