# Supplementary figures and images for: Gain and loss of an intron in a protein-coding gene in Archaea: the case of an archaeal RNA pseudouridine synthase gene
Source: BMC Evol Biol. 2009 Aug 11;9:198. doi: 10.1186/1471-2148-9-198 (PMC2738675; doi:10.1186/1471-2148-9-198)

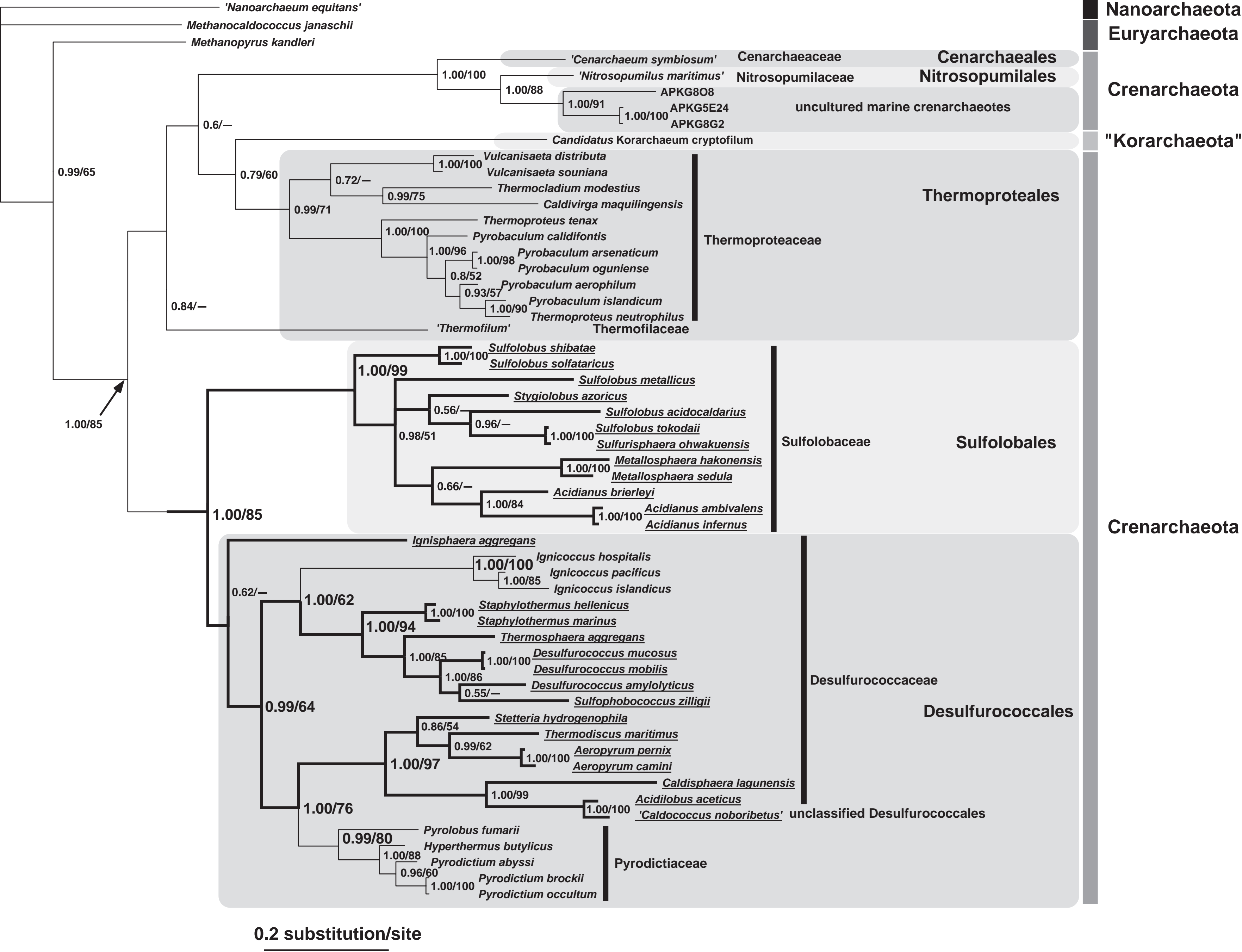

Supplement: Additional file 4 — Bayesian phylogenetic tree of crenarchaeal Cbf5 protein. Crenarchaeal Cbf5 sequences, which are not included in Figure 2, are included. [file 1471-2148-9-198-S4.pdf]

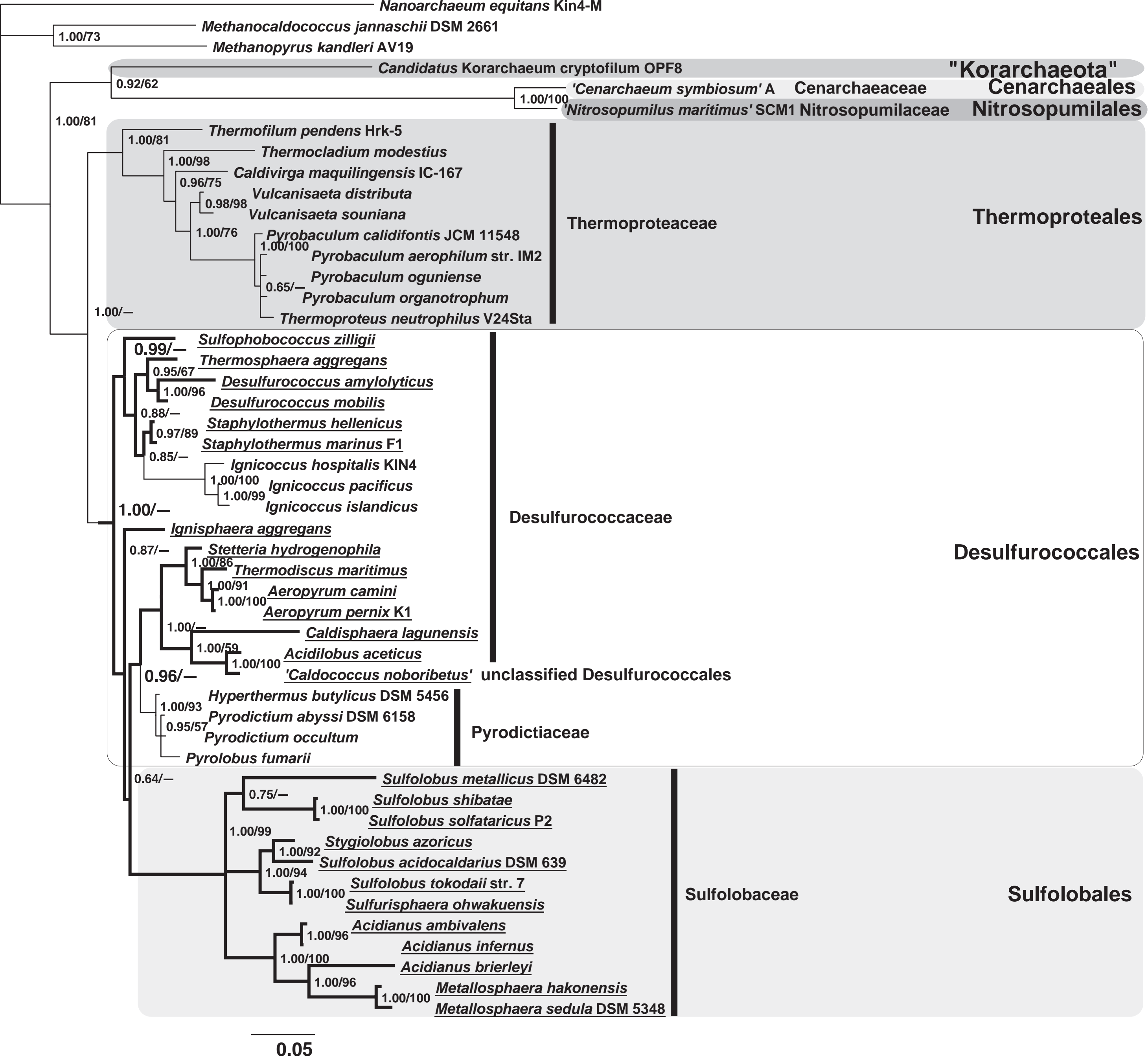

Supplement: Additional file 8 — Bayesian phylogenetic tree of the crenarchaeal 16S rRNA. This is for comparison with cbf5 tree. [file 1471-2148-9-198-S8.pdf]
