# Supplementary material for: Gain and loss of an intron in a protein-coding gene in Archaea: the case of an archaeal RNA pseudouridine synthase gene
Source: BMC Evol Biol. 2009 Aug 11;9:198. doi: 10.1186/1471-2148-9-198 (PMC2738675; doi:10.1186/1471-2148-9-198)
Supplement: Additional file 5 — The results of statistical tests of analysis 1. Comparisons of statistical supports of each grouping concerning the phylogeny of the outgroups of Sulfolobales and Desulfurococcales. [file 1471-2148-9-198-S5.pdf]

Additional file 5. The results of statistical tests of analysis 1: Comparisons of statistical supports of each grouping concerning the phylogeny of the outgroups of Sulfolobales and Desulfurococcales.

| Grouping | AU | NP | KH |
|---|---|---|---|
| **(6)**+**(7)**+**(8-11)** | 0.938 | 0.799 | 0.907 |
| (2)+(3)+(4)+(5) | 0.931 | 0.647 | 0.778 |
| (2)+(3)+(4) | 0.792 | 0.432 | 0.51 |
| (2)+(3) | 0.757 | 0.488 | 0.602 |
| (4)+(5) | 0.658 | 0.494 | 0.49 |
| **(6)**+**(7)** | 0.616 | 0.376 | 0.482 |
| **(7)**+**(8-11)** | 0.514 | 0.437 | 0.575 |
| (2)+(4) | 0.49 | 0.156 | 0.398 |
| (2)+(4)+(5) | 0.469 | 0.179 | 0.317 |
| (3)+(4) | 0.436 | 0.226 | 0.478 |
| (2)+(3)+(5) | 0.408 | 0.188 | 0.485 |
| **(6)**+**(8-11)** | 0.338 | 0.271 | 0.425 |
| (4)+(5)+**(6)**+**(7)**+**(8-11)** | 0.324 | 0.151 | 0.126 |
| (3)+(4)+(5) | 0.301 | 0.083 | 0.382 |
| (5)+**(6)**+**(7)**+**(8-11)** | 0.259 | 0.104 | 0.203 |
| (2)+(3)+(4)+**(6)**+**(7)**+**(8-11)** | 0.242 | 0.093 | 0.206 |
| (2)+(3)+**(6)**+**(7)**+**(8-11)** | 0.237 | 0.131 | 0.195 |
| (2)+(4)+**(6)**+**(7)**+**(8-11)** | 0.204 | 0.038 | 0.133 |
| (2)+(5) | 0.202 | 0.096 | 0.225 |
| (2)+(3)+**(6)**+(5) | 0.197 | 0.065 | 0.133 |
| (2)+**(6)**+**(7)**+**(8-11)** | 0.194 | 0.164 | 0.14 |
| (2)+(3)+(5)+**(6)**+**(7)**+**(8-11)** | 0.166 | 0.08 | 0.21 |
| (4)+**(6)**+**(7)**+**(8-11)** | 0.15 | 0.048 | 0.222 |
| (3)+(5) | 0.14 | 0.081 | 0.285 |
| (3)+(4)+(5)+**(6)**+**(7)**+**(8-11)** | 0.136 | 0.06 | 0.088 |
| (3)+**(6)**+**(7)**+**(8-11)** | 0.133 | *0.027* | 0.116 |
| (3)+(5)+**(6)**+**(7)**+**(8-11)** | 0.1 | *0.02* | 0.076 |
| (2)+(5)+**(6)**+**(7)**+**(8-11)** | 0.099 | *0.021* | 0.105 |
| (3)+(4)+**(6)**+**(7)**+**(8-11)** | 0.066 | *0.012* | 0.073 |
| (5)+**(8-11)** | 0.059 | *0.022* | *0.037* |
| (2)+(4)+**(6)**+**(7)** | 0.054 | *0.001* | *0.004* |
| (4)+**(7)**+**(8-11)** | 0.053 | *0.014* | 0.08 |

| | | | |
|---|---|---|---|
| (2)+(4)+(5)+**(6)**+**(7)**+**(8-11)** | *0.048* | *0.017* | 0.104 |
| (2)+(4)+**(8-11)** | *0.043* | *6.00E-05* | *0.004* |
| (3)+(4)+**(7)**+**(8-11)** | *0.042* | *0.002* | *0.004* |
| (2)+**(6)**+**(7)** | *0.038* | *0.001* | *0.038* |
| (5)+**(6)**+**(8-11)** | *0.037* | *0.005* | *0.053* |
| (2)+(3)+(4)+(5)+**(6)** | *0.033* | *0.008* | 0.093 |
| (2)+(3)+(4)+**(8-11)** | *0.032* | *5.00E-04* | *0.023* |
| (2)+(3)+(4)+**(6)** | *0.031* | *0.002* | *0.037* |
| (4)+**(8-11)** | *0.031* | *0.003* | *0.042* |
| (2)+(3)+(4)+(5)+**(7)** | *0.03* | *0.007* | *0.041* |
| (2)+(3)+(4)+**(6)**+**(7)** | *0.03* | *0.001* | *0.042* |
| (2)+**(6)** | *0.029* | *0.002* | *0.038* |
| (2)+**(8-11)** | *0.029* | *0.001* | *0.028* |
| (3)+(4)+**(6)**+**(7)** | *0.029* | *0.001* | *0.006* |
| (4)+**(6)** | *0.027* | *0.001* | *0.035* |
| (2)+(3)+(4)+(5)+**(6)**+**(7)** | *0.026* | *0.003* | *0.036* |
| (2)+(3)+(5)+**(6)**+**(7)** | *0.026* | *0.005* | *0.034* |
| (2)+(3)+**(7)**+**(8-11)** | *0.026* | *3.00E-04* | *0.004* |
| (3)+(4)+**(6)**+**(8-11)** | *0.025* | *0.004* | *0.008* |
| (2)+(3)+**(8-11)** | *0.025* | *0.002* | *0.003* |
| (2)+(5)+**(6)**+**(7)** | *0.025* | *1.00E-04* | *0.009* |
| (2)+**(6)**+**(8-11)** | *0.024* | *0.001* | *0.027* |
| (5)+**(7)**+**(8-11)** | *0.023* | *0.006* | *0.043* |
| (2)+(3)+(4)+**(7)**+**(8-11)** | *0.023* | *0.001* | *0.029* |
| (2)+(3)+**(6)** | *0.023* | *0.001* | *0.022* |
| (5)+**(6)** | *0.022* | *0.002* | *0.039* |
| (2)+(3)+(4)+(5)+**(7)**+**(8-11)** | *0.021* | *0.005* | *0.092* |
| (2)+(3)+(4)+**(7)** | *0.021* | *0.003* | *0.011* |
| (3)+(5)+**(7)**+**(8-11)** | *0.021* | *0.002* | *0.012* |
| (2)+(3)+(4)+(5)+**(8-11)** | *0.02* | *0.003* | *0.049* |
| (2)+(3)+(4)+(5)+**(6)**+**(8-11)** | *0.02* | *0.005* | *0.049* |
| (2)+(3)+(4)+**(6)**+**(8-11)** | *0.02* | *0.001* | *0.041* |
| (4)+(5)+**(7)** | *0.02* | *2.00E-04* | *0.011* |
| (4)+(5)+**(7)**+**(8-11)** | *0.019* | *0.001* | *0.026* |
| (2)+(3)+(5)+**(7)**+**(8-11)** | *0.018* | *0.001* | 0.052 |
| (4)+**(7)** | *0.017* | *0.002* | 0.066 |
| (3)+(4)+(5)+**(7)** | *0.017* | *0.001* | *0.019* |
| (3)+(5)+**(6)**+**(8-11)** | *0.017* | *2.00E-04* | *0.011* |
| (2)+(4)+**(7)** | *0.017* | *0.001* | *0.004* |
| (2)+**(7)** | *0.016* | *0.005* | *0.017* |
| (2)+(4)+**(7)**+**(8-11)** | *0.016* | *0.001* | *0.014* |
| (3)+(4)+**(6)** | *0.016* | *0.001* | *0.003* |
| (3)+(5)+**(7)** | *0.016* | *5.00E-04* | *0.018* |
| (5)+**(6)**+**(7)** | *0.015* | *0.001* | *0.039* |
| (5)+**(7)** | *0.015* | *0.001* | *0.036* |
| (4)+**(6)**+**(7)** | *0.015* | *0.001* | 0.066 |
| (3)+(4)+(5)+**(6)**+**(8-11)** | *0.015* | *0.001* | *0.008* |

| | | | |
|---|---|---|---|
| (3)+**(7)** | *0.015* | *1.00E-04* | *0.004* |
| (3)+(4)+(5)+**(7)**+**(8-11)** | *0.014* | *0.001* | *0.015* |
| (3)+**(6)** | *0.014* | *0.002* | *0.02* |
| (2)+(5)+**(7)** | *0.014* | *2.00E-04* | *0.005* |
| (3)+**(6)**+**(8-11)** | *0.014* | *3.00E-04* | *0.016* |
| (3)+(4)+(5)+**(6)**+**(7)** | *0.013* | *4.00E-04* | *0.03* |
| (2)+(5)+**(6)**+**(8-11)** | *0.013* | *8.00E-05* | *0.004* |
| (2)+**(7)**+**(8-11)** | *0.012* | *0.006* | *0.02* |
| (4)+**(6)**+**(8-11)** | *0.012* | *0.001* | *0.019* |
| (3)+(4)+(5)+**(6)** | *0.012* | *4.00E-04* | *0.023* |
| (3)+**(7)**+**(8-11)** | *0.012* | *4.00E-04* | *0.016* |
| (2)+(3)+(5)+**(6)**+**(8-11)** | *0.011* | *3.00E-04* | *0.016* |
| (2)+(3)+(5)+**(8-11)** | *0.01* | *3.00E-04* | *0.023* |
| (3)+(5)+**(6)** | *0.009* | *1.00E-04* | *0.039* |
| (3)+(4)+(5)+**(8-11)** | *0.009* | *0.001* | *0.019* |
| (3)+(5)+**(6)**+**(7)** | *0.009* | *3.00E-04* | *0.032* |
| (2)+(3)+**(6)**+**(8-11)** | *0.009* | *4.00E-04* | *0.006* |
| (2)+(3)+(5)+**(7)** | *0.008* | *5.00E-04* | *0.034* |
| (3)+(4)+**(7)** | *0.007* | *2.00E-04* | *0.011* |
| (2)+(5)+**(7)**+**(8-11)** | *0.006* | *2.00E-04* | *0.011* |
| (3)+(5)+**(8-11)** | *0.006* | *3.00E-05* | *0.023* |
| (4)+(5)+**(6)**+**(7)** | *0.005* | *2.00E-04* | *0.006* |
| (3)+**(6)**+**(7)** | *0.004* | *1.00E-04* | *0.006* |
| (2)+(5)+**(6)** | *0.004* | *3.00E-05* | *0.003* |
| (4)+(5)+**(6)** | *0.003* | *7.00E-05* | *0.005* |
| (2)+(3)+**(7)** | *0.003* | *1.00E-04* | *0.006* |
| (2)+(4)+**(6)**+**(8-11)** | *0.003* | *8.00E-05* | *0.005* |
| (2)+(4)+**(6)** | *0.002* | *3.00E-06* | *0.004* |
| (2)+(4)+(5)+**(7)** | *0.002* | *1.00E-04* | *0.002* |
| (2)+(3)+**(6)**+**(7)** | *0.001* | *2.00E-05* | *0.005* |
| (2)+(4)+(5)+**(7)**+**(8-11)** | *0.001* | *1.00E-04* | *0.007* |
| (4)+(5)+**(6)**+**(8-11)** | *6.00E-05* | *4.00E-06* | *0.007* |
| (4)+(5)+**(8-11)** | *4.00E-05* | *5.00E-06* | *0.007* |
| (3)+(4)+**(8-11)** | *3.00E-05* | *2.00E-06* | *0.006* |

The member of each category appeared in Figure 3. The categories belonging to either Sulfolobales or Desulforococcales are shown with boldface letters. Branching order within each category is as shown in Figure 3. The category (1) is treated as the outgroup. Among all possible 10,345 tree topologies, the log-likelihoods estimated by TREE-PUZZLE 5.2 under WAG+I+G model were compared. Among these tree topologies, top 2000 tree topologies were selected and used for further analyses described in the “Materials and methods” in main text. Each value less than 0.05 is indicated with italic letters.
