# Supplementary material for: Gain and loss of an intron in a protein-coding gene in Archaea: the case of an archaeal RNA pseudouridine synthase gene
Source: BMC Evol Biol. 2009 Aug 11;9:198. doi: 10.1186/1471-2148-9-198 (PMC2738675; doi:10.1186/1471-2148-9-198)
Supplement: Additional file 6 — Alignment of archaeal Cbf5 sequences used in the analysis for Figure 2. #; selected positions for the analysis. [file 1471-2148-9-198-S6.pdf]

Gblocks 0.91b Results

Number of sequences: 36
Alignment assumed to be: Protein
New number of positions: 202

```
                          10        20        30        40        50        60
                 =========+=========+=========+=========+=========+=========+
P_arsenaticum    ------------------------------------------------------------
P_aerophilum     -----------------------MKCPSREVFSKFEESTNPQWGKPPSQRSTEEYIKYSL
P_calidifontis   -----------------------MKCGSREVFVKLEEGTNPQWGKPPSSRSAEEHIRYSF
T_tenax          ------------------------------------------------------------
V_souniana       ------------------------------------------------------------
T_modestius      ------------------------------------------------------------
K_cryptofilum    ------------------------MIDEVEEIAVKSAYEPGPYGYRPQERPIFLYLDHGL
N_equitans       MLCLSLMRIFCSKCLEEQECPLPWELEKYEIWVKKEAETNEKWGEDPYNRPIERLLKYSV
M_kandleri       ----------MSGDKDRRLP----FDRDREMITKAEVETDPRYGCPPEERPIEEYIMKGV
M_janaschii      -------MILLEKTQEKKIN------DKEELIVKEEVETNWDYGCNPYERKIEDLIKYGV
Thermofilum      ------------------------------------------------------------
S_hellenicus     ------------------------------------------------------------
D_mucosus        ------------------------------------------------------------
D_amylolyticus   ------------------------------------------------------------
T_aggregans      ------------------------------------------------------------
I_pacificus      ------------------------------------------------------------
I_islandicus     ------------------------------------------------------------
I_hospitalis     ----------VKEALEWYEEMHRKCDFRGKWVIREEAETDPKYGKEPWKRDVKELVELGV
A_aceticus       ------------------------------------------------------------
C_lagunuensis    ------------------------------------------------------------
A_pernix         --MAEEAVDARGEGARFIEKVKSICGNTSRILYKYDEPTDPRYGYLPHERPLDVYLRYGM
S_hydrogenophil  ------------------------------------------------------------
T_maritimus      ------------------------------------------------------------
P_abyssi         ------------------------------------------------------------
H_butylicus      ------------------------------------------------------------
P_fumarii        ------------------------------------------------------------
A_ambivalens     ------------------------------------------------------------
A_brierleyi      ------------------------------------------------------------
S_tokodaii       -----------MEIYDFIYKIDSFCNYSNNWNIIQDSYTNEKYGYFADKRPIEVLIKNSI
S_acidocaldariu  ------------------------------------------------------------
S_solfataricus   -----------MILNDFIYKIDNFCGYKNEWKIRKDSETSDKYGYYPEKRPIEIHIKNSI
M_haknonensis    ------------------------------------------------------------
S_metallicus     ------------------------------------------------------------
I_aggregans      ------------------------------------------------------------
N_maritimus      ----------------------MTLKQLENLIEVDQDITDDAYGTYYDKRTIEQLLNYGI
C_symbiosum      ----------------------LTLAQLSGLRVIDQDVTDDAHGTYHDKRSVEQLLEYGI
```

```
                         70        80        90       100       110       120
                 =========+=========+=========+=========+=========+=========+
P_arsenaticum    -------------VAAWVKKILG-VERAGHAGTLDPKVSGVLPIAVAEGTKVLMALSRSD
P_aerophilum     VILDKPRGPSSHEVAAWVKKILG-VERAGHAGTLDPKVSGVLPIAIAEGTKVLMALSRSD
P_calidifontis   LILDKPRGPTSHEVAAWVKKILG-VERAGHSGTLDPKVSGVLPVAVAEGTKVLMALSRAD
T_tenax          --------------VAWLKRILN-VEVAGHSGTLDPNVSGVLPVAIAEGTKALMALSRAD
V_souniana       --------------AAWIKKMLN-LDRAGHSGTLDPRVSGVLPIALGESTKAMPAINNLD
T_modestius      --------------VSWIKKMMG-LERAGHAGTLDPRVSGVLPVALGDSTKVLQGISHED
K_cryptofilum    IILDKPKGPSSHEVTERVKRILEYPGKVGHCGTLDPKVSGVLPIVLGNATKLSKFISGYD
N_equitans       INLDKPSGPTSHQVVAWVRDIVG-V-KAGHGGTLDPKVTGVLPIAIGEATKVLQTLLIAG
M_kandleri       INLDKPAGPTSHEVVAWVKEIFG-LSKAGHGGTLDPKVTGVLPIALEKATKIIQTLLPAG
M_janaschii      VVVDKPRGPTSHEVSTWVKKILN-LDKAGHGGTLDPKVTGVLPVALERATKTIPMWHIPP
Thermofilum      -------------VVAWLRKLLG-IERIAHAGTLDPKVSGVLPITLNNAVRVLPVLLKED
S_hellenicus     --------------VAWIKKMFE-LERAGHGGTLDPKVTGVLPVALANATKVIGNVIHTI
D_mucosus        --------------VAWVKKMFG-VEKAGHGGTLDPKVTGVLPVGLANSTKVIGNVIHSV
D_amylolyticus   --------------VAWVKKMFN-LEKAGHGGTLDPKVTGVLPIGLANSTKVIGNVIHSV
T_aggregans      --------------VAWVKKLFN-IDKAGHGGTLDPKVTGVLPVGLDNSTKVIGNVVHSV
I_pacificus      --------------VAWIKKMFN-LEKAGHGGTLDPKVTGVLPVALAEATKVIHYVMLSG
I_islandicus     --------------VAWIKRMFN-LDKAGHGGTLDPKVTGVLPVAFKEATKVIHYVMLSG
I_hospitalis     VNLDKPPGPTSHEVVAWIKRMFG-LERAGHGGTLDPKVTGVLPVALAEATKVIHYVMLSG
A_aceticus       --------------TAWVKRLLG-VKRAGHGGTLDPKVTGVLPVALDHMTKIMGSVVHSR
C_lagunuensis    --------------VAWIKRLLN-VDKAGHAGTLDPRVSGVLPVTLGHMTKIIGYITHSN
A_pernix         IVVDKPPGPTSHEVVAWIKRMLG-VSRAGHGGTLDPKVTGVLPVALERMTRIIGTVMHSS
S_hydrogenophil  --------------VAWVKRMFN-VPRAGHGGTLDPRVTGVLPVALHRMTRVVGSVVHSS
T_maritimus      --------------VAWIKRMFN-IKRAGHGGTLDPKVTGVLPVALQRMTRIVGSVIHSD
P_abyssi         --------------VAWVKRMFG-LSKAGHGGTLDPKVTGVLPVALEEATKVIGLVVHTG
H_butylicus      --------------VAWIKRMFG-LSKAGHGGTLDPKVTGVLPVALEEGTKVIGLVVHTG
P_fumarii        --------------VAWIKRMFG-LSKAGHGGTLDPKVTGVLPVALEEATKIIGMVVHTP
A_ambivalens     --------------AFWIKKMFN-LSKVGHGGTLDPKVSGVLPIGLENATKLMTYITKAG
A_brierleyi      --------------AFWVKKMFN-INKVGHGGTLDPKVTGVLPIGLENATKIMNYVTEAG
S_tokodaii       INADKPPGPTSHEVAYWIKQMFK-VSKAGHGGTLDPKVTGVLPIGLENATKLMSYISSSG
S_acidocaldariu  --------------AYWVKKLFN-ITKAGHGGTLDPKVTGVLPIGLENATKLMNYISKSG
S_solfataricus   INLDKPPGPTSHEVAYWVKKMLN-VTKAGHGGTLDPKVTGVLPIGIENATKIMNYISKGG
M_haknonensis    --------------AFWVKTMFG-LPRVGHGGTLDPKVTGVLPIGLGKATRVMNLITKSG
S_metallicus     --------------AFWIKQRLS-LNRVGHGGTLDPKVTGVLPVGLDNATKIMHYVSRST
I_aggregans      --------------VAWIKKMFN-IPKAGHGGTLDPKVTGVLPVALDRATRVIGILMHSY
N_maritimus      ILLDKPPGPTSHETVAWTKRILK-LPKIGHSGTLDPQVSGVLPLGLGEATKALGVLLFGP
C_symbiosum      VLLDKPPGPTSHEAVAWAKRILE-IPKAGHSGTLDPQVSGVLPLGLGEATKALGVLLIGP
                                 ######    ##################################
```

```
                         130       140       150       160       170       180
                 =========+=========+=========+=========+=========+=========+
P_arsenaticum    KVYVAVAKFHGDVDEERLRAVLREFQGEIYQKPPLRSAVKRQLRTRRVFSLELLELEG--
P_aerophilum     KVYVAVAKFHGDVDEDKLRAVLQEFQGVIYQKPPLRSAVKRQLRTRRVYSLDLLELDG--
P_calidifontis   KVYIAVAKFHGDVDVENLRRVLQELQGEIYQKPPLRSAVKRQLRTRRVYSLELLELDG--
T_tenax          KTYVAVAKFHGDVDEAKLREVLAYFTGAIYQRPPLRSAVKRQLRVRHVYSLELLELDG--
V_souniana       KEYIMVMKLHGDVDDGKLRAVLREFTGAIYQRPPLRSAVKRQLRVKHVYELELLERDG--
T_modestius      KEYIAVMTLHGDASDERIREVLAEFTGEIYQRPPVKSAVKRQLRTRRVYSLKMLERDG--
K_cryptofilum    KEYVGTLYLHGDVPIDELKGALDKFTGPIFQRPPVKSAVKRSLRVRRVYSIELLSSEG--
N_equitans       KEYVALMHLHKEVSEKDIIKVMSKFVGTIIQTPPLRSAVKKRPRKKKVYCIKIIEIDG--
M_kandleri       KEYVTIMHLHGDVDEEELERVVKEFEGTILQRPPLRSAVKRRVRPKKIYYIDILEIDG--
M_janaschii      KEYVCLMHLHRDASEEDILRVFKEFTGRIYQRPPLKAAVKRRLRIRKIHELELLDKDG--
Thermofilum      KEYVCVMRLHGDVDPERLERVVSMFKGRIYQRPPLRSAVKREVRIRQIYDIRLLEFNE--
S_hellenicus     KEYVMVIQLHAPVDNDRLRKVLKYFTGVIYQRPPLRSSVKRVIRTRRIHYIDLLEHS--D
D_mucosus        KEYVMVIQLHGDVAEQDLRRVAGYFRGEIYQRPPLRSSVKRAIRVRRIHEIEVLEVR--G
D_amylolyticus   KEYVMVIQLHGDARESSVKMVVEYFKGDIYQKPPLRSSVKRSIRVRRIYEIELLEIR--D
T_aggregans      KEYVMVIQFHGDVEIGKLVEALKYFTGEIYQRPPLRSSVKRVLRTRRIHEIELLDFK--D
I_pacificus      KEYVMVIQFHDAVKEQDVIENLKYLVGEIYQRPPLRSSVKRQLRTKKVYYIHLIEFYPER
I_islandicus     KEYVMVIQFHDAFNEKDVIENLKYLVGEIYQRPPLRSSVKRQLRTKKVYYIHLIEFYPER
I_hospitalis     KEYVMVIQFHDAVKEEEVIENLKYLVGEIYQRPPLRSSVKRQLRTKKVYYIHVLEFWPER
A_aceticus       KAYVCVMQLHGDVSEKKLREVAEEFTGTIYQRPPVRSNVKRALRTRRIFSLEVLEVRG--
C_lagunuensis    KTYVCVMQLHKDVDQNELKRVINDFIGTIYQKPPIRSHVKRTLRTRKIFNITINEIVD--
A_pernix         KEYVCVMQLHRPVEEDRLREVLKLFEGEIYQKPPLRSSVKRALRTRRVFRIELLEYTG--
S_hydrogenophil  KEYVCVMQLHRPVEEERLREALRLFEGRIYQRPPLRSSVKRSLRVKRVDEIELLEYDG--
T_maritimus      KEYVCVMQLHAPVEEERLREVLNEFEGVIYQRPPLRSSVKRALRKKRVYKIELLEYTG--
P_abyssi         KEYTCVMQLHHPVPEQDLRRALEMFVGEIYQRPPLRSSVKRSLRTKKIYEIELLEYNG--
H_butylicus      KEYMCVMQLHRPVPEEELRRAINMFVGEIYQRPPLRSSVKRSLRLKKIYEIELLEYNG--
P_fumarii        KEYICVMQLHEPVEEKKLLEAIKVFTSTIYQRPPLRSSVKRSLRTKTIYEIELLEYTG--
A_ambivalens     KEYICVMETHEKVDLSIVKKIAEEFKGTIYQRPPVRSSVKRRLRTRKVFDIEVLEGKDD-
A_brierleyi      KEYICVMQVHCDIEKDLLKSIIEKFKGKIYQRPPVRSSVKRRLRFRTVNEIELLETYN--
S_tokodaii       KEYVCLMQVHCDFNIDELKQIISKFIGIIYQKPPVRSSVKRRTRKKKIYDIEILDTDK--
S_acidocaldariu  KEYICLLQMHCNVDQKELKEIISQFVGEIYQKPPVRSSVKRRIRKRRIYAIDILDMQD--
S_solfataricus   KEYVCVMQVHCEYNKEELAKIISSFKGEIYQRPPVRSSVKRRLRIRRIYDIEILDMDK--
M_haknonensis    KEYICLMEVHCEFSEDKLREVSKQFVGTIYQKPPVRSSVKRRVRKREIYSLDLLEISG--
S_metallicus     KEYVCVMQVHSDFEKESLKSWIMKFKGKIYQRPPVRSSVSRKLRTRKIYDIEVTEIED--
I_aggregans      KEYVGVMELHGDVEPEKVIETMKMFVGKIYQRPPLRSSVKRSLRIREIYSIDVLEIEN--
N_maritimus      KEYHALGRVHSLPSKEKLHEVIESLTGEIYQKPPQRSAVVRQTRTRTIYEFEVLEQKE--
C_symbiosum      KEYVAVGRFHALQDSEKLGELARLFTGPIHQKPPQRSSVLRRTRVKTIHEIEVLEQKE--
                 #######################################################
```

```
                         190       200       210       220       230       240
                 =========+=========+=========+=========+=========+=========+
P_arsenaticum    -RYAVIKMHVEAGTYARKIIHDIGEVLGVGANMRELRRVAVTCFTED-E-AVTLQDVADA
P_aerophilum     -RYAVIKMHVEAGTYARKIIHDIGEVLGVGANMRELRRIAVSCYTED-E-AVTLQDIADA
P_calidifontis   -RYAVLKMHVEAGTYARKLIHDLGEILGVGANMRELRRVAVSCFTED-E-AVTLQDLADA
T_tenax          -RYALLRMHVEAGTYARKLIHDIGEVLGVSANMRELRRVGVGCFTEE-E-AFTLQDIADA
V_souniana       -KYALIRMNVESGTYARKLAYDIGEVLGVGANMRELRRIRVGCFTEK-E-AITLQDLKDA
T_modestius      -RYVLLDARVEAGTYIRKLCYDVGEVLGVGANMRELRRVRVGCFGED-E-AVTLHDLADA
K_cryptofilum    -RFHKLRVRVESGTYIRKLFFDIGEFLGVGGSMRDLRRIRSGIFTEK-D-CVTLEDIKDA
N_equitans       -KDVLFRVSTQGGVYIRKLIHDIGVKLGVGAHMQELRRIKSGPFHEN-N-SVYLQDIVDS
M_kandleri       -RDVLMRVGCQAGTYIRKLCHDIGEALGVGAHMAELRRTRTGPFSEE-N-AVTLHDVKDA
M_janaschii      -KDVLFRVKCQSGTYIRKLCEDIGEALGTSAHMQELRRTKSGCFEEK-D-AVYLQDLLDA
Thermofilum      -RTALLHVWCEAGTYMRKLCHDIGEILGVGAHMQELRRIRSGSLYEDRN-CSTMHDVVDA
S_hellenicus     -RYVLVRVGCEAGTYMRKLAHDIGLLLGVGAHMRELRRTRTGPYKEDET-LVRMQEVSEA
D_mucosus        -RFALIRVLSDPGTYMRKLAHDIGLLLGVGAHMRELRRTRTGPYREDET-LVRLQDVSEA
D_amylolyticus   -RFILVRVLSDPGTYMRKLAHDIGLMLGTGAHMRELRRTRTGPYREDET-LVRLQDISEA
T_aggregans      -RFALVRASCDPGTYMRKLAHDIGLYTGVGAHMRELRRTRTGPYREDET-LVKLQDVSEA
I_pacificus      -RMALVRVGSESGTYMRKIAHDLGLLLGTGAHMRELRRTRSGPFHEEYN-LVRMQELSEA
I_islandicus     -RMVMVRVGSESGTYMRKIAHDLGLLMGIGAHMRELRRTRSGPFHEGYN-LVTMQELSEA
I_hospitalis     -RMALVRVGSESGTYMRKLAHDLGLLVGTGAHMRELRRTRSGPFHEDWN-LVRMQDLSEA
A_aceticus       -RLALLRVESDPGTYMRKLCWDMGLVLGVGAHMRELRRIKTGPFDEDHN-LVTLQELSEA
C_lagunuensis    -RKVLLTIESEAGTYMRKLCWDIGLILGIGAHMRELRRIKTGPFNEDYY-LVTMQDLTEA
A_pernix         -KYALLRVDCEAGTYMRKLCWDIGLVLGVGAHMRELRRIRTGPFSEDSGLMVRLDDVAYA
S_hydrogenophil  ERYALLRIACEAGTYMRKICWDVGLVLGVGAHMRELRRTRTGPFSEARG-LVTLQEVSEA
T_maritimus      -KYALLHIWSEAGTYMRKICWDVGLVLGTGAHMRELRRIRTGPFTEERG-LVRMQDIAYA
P_abyssi         -RYALMRVFCEAGTYMRKLCHDVGLILGVGAHMRELRRTKSGPFREQHG-LVRLQDLSEA
H_butylicus      -RYALMRVFSEAGTYMRKLCHDIGLILGVGAHMRELRRTRSGPFRERNG-LVRLQELSEA
P_fumarii        -RYALMRVLCDPGTYMRKLCHDIGLYLGVGAHMRELRRTKSGPFREAYG-LVKLQDLSEA
A_ambivalens     -KLFLLRISSEPGTYMRKICHDMGIILGTGAHMRELRRIRSGIFTES-G-LFTLQEVSEA
A_brierleyi      -RMALLRISSEPGTYMRKICHDAGILLGCGAHMRELRRIRSGIFTEK-N-LVTLQEVSEA
S_tokodaii       -RFILLRISSDPGTYMRKLCHDIGVILGCGAHMRELRRIRSGIFTEK-N-LVTLQEISEA
S_acidocaldariu  -KLILLRVQSDAGTYMRKLCHDVGVIAGCGSHMRELRRIRSGIFTEKTN-MVTLQEVSES
S_solfataricus   -KLVLLRVNCDSGTYMRKLCHDIGIIYGCGAHMRELRRTRSGIFTESTN-LVKLHELSEA
M_haknonensis    -RRVLMRISSEPGTYMRKICHDMGILLGCGAHMRELRRTKSGIFKED-T-LVTLQQVSEA
S_metallicus     -RLVLMKVISEHGTYMRKLCHDIGILSGYGAHMRELRRTRTGIFDER-N-LVTMHEFSEA
I_aggregans      -RRVLFRVKCQSGTYVRKLCHDIGLLLGVEAHMRELRRIGVAHFTENRD-IVTLHEVSEA
N_maritimus      -RLLLTRVLCEAGTYIRKLYYDLGEILGPGATMIELRRTRVDQFRETDG-LVTLHELANA
C_symbiosum      -RLVLMRVSCEAGTYIRKLLYDMGEVVGSGASMIELRRTRVDHFTEGSG-LVTMHELAEA
                  #############################################    ##########
```

```
                         250       260       270       280       290       300
                 =========+=========+=========+=========+=========+=========+
P_arsenaticum    YYIWKKYGDDTYLRSVLLPIEEIARHLPKIWVRDSAVDAVCHGAPLAAPGISKFEVPFSK
P_aerophilum     YYIWKHYGDDTYLRRVLLPIEEIARHLPKIWVRDSAVDAICNGAPLAAPGISKFETPFSK
P_calidifontis   YYIWKKYGDDTYLRRVLLPIEEIARPLPKIWVRDSAVDALCNGAPLAAPGVAKFEHPFSR
T_tenax          VYIWRNYGDDTVLRQIVKPIEEIARGVAKIWIKDGAVDAVCHGAPLAAPGVVKFEEPFQR
V_souniana       YTLYRDYGIEDLLRTYVKSVEYMVGHLPKVWVRDSAVDAICHGAALAVPGIVKLTNNIKR
T_modestius      LYLWKQYGDESLLRRYVKPVEYMVKHLPRIFIRDSAVDSVAHGAALAAPGVAKIEDGVER
K_cryptofilum    YDSWRESGDESKIRKVILPLEEAVRHLPKIYVKDSAVASLTHGASLKVKGICSLSRGIKK
N_equitans       LYFWKEEGNEEYIRKVFLPVEEAVKHLKKIYILDSAVAAIVHGANLAVPGIAKLYSNIKK
M_kandleri       YEFWKEEGWEEPLRHVVRPMEEGLEHLPRIEIRDTAVDAICHGANLAAPGIVRVEKGIQP
M_janaschii      YVFWKEDGDEEELRRVIKPMEYGLRHLKKVVVKDSAVDAICHGADVYVRGIAKLSKGIGK
Thermofilum      YYIWKERGIDHFLRQVFLPVEAAIQHLPKVWIRDSAVDAVCHGAPLAVPGIVKLEGGIKV
S_hellenicus     LYLWRNKGDERYLRKIILPVETAIAHLPKIIIRDTAVDAIAHGAHLAVPGIARLTRDVAP
D_mucosus        LLLWRSNGDERYLRRIVLPVETSIAHLPKIMILDTAVDAIAHGANLAAPGVARLTRNIAK
D_amylolyticus   LALWRSSGDERYIRRIILPVETSIAHLPKIMILDTAVDAIAHGANLAVPGIARLTSNVER
T_aggregans      LYLWRVEKDERMLRRVILPVETAVTHLPKIVINDLAVDAIAHGASLAAPGVVRLTDNVSA
I_pacificus      KFLWDHLKDDSLLKKYIMPCEYAVCHMQKIMVGDGAVDAIAHGAHVAAPGVAALTDGIKK
I_islandicus     KFLWEEKKDDSLLRKYVMPCEYSVCHMQKVMVMDGAVDAIAHGAHVAAPGVAALTDGIRK
I_hospitalis     KFLYDNYGDDSLLKKYIMPCEVATCHMPKIMIKDGAVDAVAHGANVSIRGVAALTDNMKK
A_aceticus       VYRFREEGKDDYLRRAVLPGEVITCELPKVVLRDSAVESVVNGAPLAVPGISLLTPDVKA
C_lagunuensis    VYRFKTEEKDDLLRKVIIPGEYSVCELPKVLVRDTAVESVINGSPLAIPGISYYNEGINR
A_pernix         VIRWREEGKDDLLRRVVIPGEYSVCHIPKVLVRDSAVESLTHGAQLAAPGVAAVEEGVEK
S_hydrogenophil  LYRLRVDGKDDLLRRVVLPGEYSVCHLPKVVVRDTAVESLVHGASLAVPGVVMLHEDIKR
T_maritimus      LIRYRDEGKEDLLRKYVLPGEYSVCHLPKVVVRDSAVESIVNGAMLAVPGIAMLHEGIEK
P_abyssi         LYRWRNEGKEDLLRKYIKPIEYAVSHLPKIVIRDTAVDAIAHGANLAVPGIVRLHEGIQR
H_butylicus      IYRWKQEGKEDLLRKYILPIEYAVSHLPKIVVRDTAVDAIAHGANLAVPGIARLHEGIKR
P_fumarii        LYRWKQEGKDDLLRKYILPMEYAVAHLKKVVIRDSAVDAIAHGAHLAVPGIARLHADIKK
A_ambivalens     LYMWKNCKDETDLRKILLPMEIGLCGIPKIILDDNAVNAIAYGATANVPGIVAYQN-FKK
A_brierleyi      LYMWKSCKDEEDLRKILIPMEMAFCGIPKIVIDDNAVDAIAYGASVMIPGIVAFQN-FKK
S_tokodaii       LYMWKNCKDESDLRKILLPMEYATCGMPKILIDDNAVDAISYGAMLTAPGIVAYQR-FRV
S_acidocaldariu  LYLYRNCKDESELRRILLPMEYGVCGIPKVIVSDTAVNAITYGAKLNLPGILAYQN-FRK
S_solfataricus   IYLYKNCKDETELRRVLIPMEFATCEIPKIVIEDSAVNALAYGAQLAVPGVVAYQN-FKK
M_haknonensis    LYLYNNCREEDELRRILIPMEYAFCGIPKIIVDDETVNSLAYGSPLMAPGIVAFQP-FKK
S_metallicus     VYMWKNCKDESFIRKIIMPMEIATCGIPKIMVDNNAVSAIAYGAKLTAPGVVGFQQ-FKK
I_aggregans      LYIWRNLGDESFLRKMILPVEYIVAFLPKIVVKDSAVDAIAHGAQLAVPGISIVAKNINR
N_maritimus      FALWEEKKDDSKLKSMIQPVEHALSELKSVVIRDSAIDAMCHGAQLAIPGILQISPSLNK
C_symbiosum      YAVWKEG-DGSRLQRIIRPVEEALAGIKAVVIRDSAVDALCHGAQLAIPGILQVSDNLRI
                 ####    ###########################################        #
```

```
                         310       320       330       340       350       360
                 =========+=========+=========+=========+=========+=========+
P_arsenaticum    GDIVAMF-----------------------------------------------------
P_aerophilum     GDLVAMFTLKGELIGIGRALVGSEEVKKMERGLVARTDRVVMRRGTYPAMWKRKAKSQSD
P_calidifontis   GDLVAYFTLKGELIGIGRALVDSEEVKKMEKGLVARTDRVVMPRGTYPPMWRRGGKSFKS
T_tenax          GDLVAYF-----------------------------------------------------
V_souniana       KSLTAIM-----------------------------------------------------
T_modestius      DRMAAIV-----------------------------------------------------
K_cryptofilum    GSIVALMTLKGELIAIGRALMDFDEMLSADSGVAASIERVIMPRDLYPPMWKTG------
N_equitans       GDLVSIHTLKGELVAIGIALMDSKEMLEKKRGIAVDIERVFMKPGLYPKMWVSQG-----
M_kandleri       GDLVAIFTLKGEAVALGVAKATWKEMLHADRGIMVDTKRVLMEPGTYPKAWGLKTPGE--
M_janaschii      GETVLVETLKGEAVAVGKALMNTKEILNADKGVAVDVERVYMDRGTYPRMWKRKK-----
Thermofilum      NSTVAIL-----------------------------------------------------
S_hellenicus     NKTVAIL-----------------------------------------------------
D_mucosus        GSTVAIL-----------------------------------------------------
D_amylolyticus   NKTVAIL-----------------------------------------------------
T_aggregans      GATVAVF-----------------------------------------------------
I_pacificus      GDVVAIV-----------------------------------------------------
I_islandicus     GDVVAIF-----------------------------------------------------
I_hospitalis     GDVVAVVSLKGELVAIAQALVSSQEALKMEKGWVAKTKRVIMKPGTYPDVWRKKKASQQE
A_aceticus       GGRVAML-----------------------------------------------------
C_lagunuensis    GDLVSLL-----------------------------------------------------
A_pernix         GGMVALMTLKGELIGLGKALASAQEMLEAERGIVVSPTRIIMERGLYPRMWKRQQAPQGA
S_hydrogenophil  GETVAVL-----------------------------------------------------
T_maritimus      GDTVALL-----------------------------------------------------
P_abyssi         GDLVALF-----------------------------------------------------
H_butylicus      GDLVALL-----------------------------------------------------
P_fumarii        GDVVAIF-----------------------------------------------------
A_ambivalens     GDLVGLI-----------------------------------------------------
A_brierleyi      GDLVGI------------------------------------------------------
S_tokodaii       KDTVAILTLKGELVAIGEADVDSQKLVDMKKGIVVKPKRVLMPRDIYPRSWKKHG-----
S_acidocaldariu  NQDVAVL-----------------------------------------------------
S_solfataricus   NDTVAVLTLKGELVATGNALMDSEELENAKKGIVVNLSRVFMQRDIYPKAWKKHES----
M_haknonensis    GDVVALI-----------------------------------------------------
S_metallicus     DDVVCVITTKGELVSVGKALMDYRRLAKVDKGEVASTDRVFIDRDVYPKHWGDKDGGSS-
I_aggregans      DDRVAIF-----------------------------------------------------
N_maritimus      GDIVGIYTQKGEAVALAEATMSGQEIQDAVKGYAFETKRIIMAPNTYPKKWRTKPSSKE-
C_symbiosum      GDLAGVYTQKGEVVALAEAEMEAEVIADATKGHAFKTKRLIMKTDTYPKSWHSKGTYKEK
                 ####
```

```
                 ==
P_arsenaticum    --
P_aerophilum     SA
P_calidifontis   GT
T_tenax          --
V_souniana       --
T_modestius      --
K_cryptofilum    --
N_equitans       --
M_kandleri       --
M_janaschii      --
Thermofilum      --
S_hellenicus     --
D_mucosus        --
D_amylolyticus   --
T_aggregans      --
I_pacificus      --
I_islandicus     --
I_hospitalis     GG
A_aceticus       --
C_lagunuensis    --
A_pernix         --
S_hydrogenophil  --
T_maritimus      --
P_abyssi         --
H_butylicus      --
P_fumarii        --
A_ambivalens     --
A_brierleyi      --
S_tokodaii       --
S_acidocaldariu  --
S_solfataricus   --
M_haknonensis    --
S_metallicus     --
I_aggregans      --
N_maritimus      --
C_symbiosum      E-
```

Parameters used Minimum Number Of Sequences For A Conserved Position: 19 Minimum Number Of Sequences For A Flanking Position: 30 Maximum Number Of Contiguous Nonconserved Positions: 10 Minimum Length Of A Block: 5 Allowed Gap Positions: None Use Similarity Matrices: Yes
Flank positions of the 6 selected block(s) Flanks: [77 82] [87 175] [182 226] [231 244] [249 291] [300 304]   New number of positions in /Users/yokobori/Desktop/Cbf5_2/list080725-5.fasta-gb: 202 (55% of the original 362 positions)
