# Supplementary material for: Gain and loss of an intron in a protein-coding gene in Archaea: the case of an archaeal RNA pseudouridine synthase gene
Source: BMC Evol Biol. 2009 Aug 11;9:198. doi: 10.1186/1471-2148-9-198 (PMC2738675; doi:10.1186/1471-2148-9-198)
Supplement: Additional file 7 — The results of statistical tests of analysis 2. Comparisons of statistical supports of each grouping concerning the phylogeny within Sulfolobales and Desulfurococcales. [file 1471-2148-9-198-S7.pdf]

Additional file 7. The results of statistical tests of analysis 2: Comparisons of statistical supports of each grouping concerning the phylogeny within Sulfolobales and Desulfurococcales .

Grouping	AU	NP	KH
**(10)**+(11)	0.831	0.697	0.829
(8)+**(9)**	0.82	0.605	0.78
**(6)**+(8)+**(9)**	0.768	0.379	0.513
**(6)**+**(7)**+**(10)**+(11)	0.746	0.276	0.041
**(7)**+**(10)**+(11)	0.723	0.285	0.577
**(7)**+(8)+**(9)**+**(10)**+(11)	0.65	0.425	0.487
**(6)**+(8)+**(9)**+**(10)**+(11)	0.569	0.276	0.423
**(6)**+**(10)**+(11)	0.559	0.155	0.414
(8)+**(9)**+**(10)**+(11)	0.523	0.274	0.423
**(6)**+**(7)**	0.396	0.129	0.376
**(6)**+**(7)**+**(10)**	0.355	0.201	0.169
**(6)**+(8)+**(10)**+(11)	0.35	0.116	0.175
(8)+**(10)**+(11)	0.348	0.15	0.22
**(6)**+**(7)**+**(9)**+**(10)**	0.329	0.058	0.194
**(7)**+(8)+**(9)**	0.325	0.048	0.314
**(6)**+**(7)**+(8)+**(9)**	0.315	0.062	0.316
(8)+(11)	0.313	0.212	0.219
**(7)**+**(10)**	0.31	0.265	0.171
**(7)**+**(9)**	0.301	0.197	0.163
(8)+**(9)**+(11)	0.289	0.161	0.171
**(9)**+**(10)**+(11)	0.287	0.078	0.163
**(7)**+**(9)**+**(10)**+(11)	0.269	0.071	0.163
**(6)**+(8)+**(9)**+(11)	0.263	0.09	0.169
**(7)**+**(9)**+**(10)**	0.245	0.057	0.194
**(6)**+(8)	0.238	0.083	0.163
**(6)**+(8)+(11)	0.234	*0.031*	0.194
**(6)**+**(10)**	0.189	*0.049*	0.151
**(6)**+**(9)**	0.187	*0.024*	0.169
**(7)**+**(6)**+**(9)**	0.187	*0.011*	0.169
**(7)**+(8)+**(10)**+(11)	0.183	*0.018*	0.163
**(6)**+7)+(8)+**(10)**+(11)	0.171	*0.036*	0.17
**(7)**+(8)+**(9)**+(11)	0.167	*0.027*	0.151
**(9)**+**(10)**	0.155	*0.02*	0.187
**(6)**+**(7)**+(8)+**(9)**+(11)	0.151	*0.012*	0.148
**(6)**+**(7)**+(11)	0.127	*0.002*	0.058
**(6)**+**(7)**+(8)	0.093	*0.004*	0.116
**(7)**+(8)	0.077	*0.009*	0.076
**(6)**+**(9)**+(11)	0.075	*2.00E-04*	*0.027*
**(6)**+**(7)**+(8)+**(10)**	0.068	*0.005*	0.057

**(6)**+**(9)**+**(10)**+(11)	0.067	*0.002*	0.074
**(7)**+(8)+**(10)**	0.067	*2.00E-04*	*0.027*
**(6)**+(8)+**(9)**+**(10)**	0.065	*0.003*	0.071
(8)+**(9)**+**(10)**	0.062	*5.00E-04*	0.071
**(6)**+(8)+**(10)**	0.055	*0.005*	0.057
**(9)**+(11)	0.054	*0.005*	0.069
**(7)**+**(9)**+(11)	*0.048*	*0.004*	0.065
**(6)**+ **(7)**+ **(9)**+**(10)**+(11)	*0.041*	*0.003*	0.107
**(6)**+(11)	*0.039*	*0.001*	0.073
**(7)**+(8)+**(9)**+**(10)**	*0.029*	*0.001*	0.075
**(7)**+(11)	*0.028*	*0.003*	0.068
**(7)**+**(6)**+(8)+**(9)**+**(10)**	*0.024*	*0.003*	0.073
(8)+**(10)**	*0.014*	*0.002*	0.098
**(7)**+(8)+(11)	*0.007*	*1.00E-04*	0.156
**(6)**+**(9)**+**(10)**	*0.005*	*2.00E*-04	0.157
**(6)**+**(7)**+(8)+(11)	*2.00E-04*	*2.00E-05*	0.154
**(6)**+**(7)**+**(9)**+(11)	*5.00E-49*	*7.00E-17*	*0.027*

The member of each category appeared in Figure 3. The groups of which members carry the intron in the *Cbf5* gene are shown with boldface letters. Each value less than 0.05 is indicated with italic letters. Branching order within each category is as shown in Figure 3. The category (1-5) is treated as the outgroup. All possible 945 tree were used for the analyses described in the “Materials and methods” in main text. Each value less than 0.05 is indicated with italic letters.
