# Supplementary material for: Gain and loss of an intron in a protein-coding gene in Archaea: the case of an archaeal RNA pseudouridine synthase gene
Source: BMC Evol Biol. 2009 Aug 11;9:198. doi: 10.1186/1471-2148-9-198 (PMC2738675; doi:10.1186/1471-2148-9-198)
Supplement: Additional file 9 — Alignment of COG1353 proteins. Sulfolobus solfataricus SSO1991, a representative of COG1353 which was predicted as a putative reverse transcriptase, and the homologs from Hyperthermus butylicus, and Ignicoccus hospitalis are included. [file 1471-2148-9-198-S9.pdf]

```
SSO1991         MSTDDNSREEFLNYKIMALLHDPPNKAWVITSRAHN------------------------
Hbut_0714       -------MSRLHMVKLAALLHDPAWKPWIVASAFPGKTGTVGRVLAKVAGKSAPAGELVK
Igni_0328       -----MTCNNLIKKKMVALLHDTPEKAWLMY-----------------------------
Igni_0463       -----MSCEELFARKAAALLHDPPEKVWASF-----------------------------
                        ..:   *  *****.. * *

SSO1991         -------LTVQLRSVRARKSHERVAKYIINQLFG------------DINSKTVDNADKLA
Hbut_0714       VEECEELMKKMKTYTIEADAHQLDAAAAAVAILDGLRDAPIIEKLILDEKGVVAEADKLA
Igni_0328       ---------------VKKK-HEEYAKERIGDVLG---------EEYLDYFKDVKAYDVIS
Igni_0463       ---------------YKHESHEKRAKDAAKEFFG---------EGFVKTLDEVKEHDRLA
                                  . *:  *      .:.                  *   * ::

SSO1991         SSIDRYLGSIVYKEYSLFRNRS--IFLKNILLSNIQRDVGNLFPKDKSKLDNLISEYKKL
Hbut_0714       ASLDRWVLTYKHREARDVKVDVGDVAYANPLDPRFKMPES-MRSIDAGVVCRYVMKLKQL
Igni_0328       STIDRWVVPS-----RMNIDRV--IRLINPFDPNFFYEID-LGQISKEKIEDYWNKLKTC
Igni_0463       STVDRWTAVG-----TEVRGRLNEVSLVNPFDPEFSYKVK-MPKIKDDAIARYRITLKSI
                :::**:                  :   * : ..:      :   .   :       *

SSO1991         LNVTNTTN--LNILKYQLFYLIYELIWIDSRYENTP-AETRNPTHTIFDHLYATAAMMNW
Hbut_0714       YGDARELP---YPLLYNLFYLLLEPLWYEVCRACIPLADTRTPTHTVFDHVYATAAMVNW
Igni_0328       LSKVSGD-----RERYHAQYVLMELLWYKTVKVPLP-ADTRFPTHTVFDHVYATASMINI
Igni_0463       LEKVEDSSKGVTRKKYHALYAFMEPAWYVEVGQPLP-ADSRFPTHTIFDHVYATAMMVNI
                   .           *:  * : *  *        * *::* ****:***:**** *:*

SSO1991         IFSLEKEAKGYLLGIDTIGVADFISKGKKTRDLWISSYLVSALLWYVITWFIEEYGPDVI
Hbut_0714       LYPGGGKPGGFLVKVDVAGIQGFISASRKTRDLWAGSWLVSALAWFTVSEAVMLLGGDIV
Igni_0328       YNNDSKKFSGFIVGIDIPGIQSFISGGRRPGDWWIRSWLISATVWYLIKELVWNLGPDVL
Igni_0463       YD-PKKNFDGFFVEVEIPEARSFLKGGRGPGDWWARGWLLSNITWRLVEELVWEVGPDIL
                      :  *::: ::     .*:. .: . * *  .:*:*   *  :   :   * *::

SSO1991         LFPSLRFNQFYAFYLLEKLRKEK--------ISEDVIDEIKELITKYIFNGDDLFEKLEI
Hbut_0714       LSPYNPANPFFMATVLEELRLAGSEYSGLVRFVEDKVERAYLWKGAANQPVIPGTIFLAL
Igni_0328       LSPSARYNPFYYATVAHRVQAVR-----------ECLDENLT---SPEQPLMPATVTLLL
Igni_0463       LFPTARYNPFYYSLIQEKIPELK-----------EEYERYLKPLGSVKNPVVPPAVSLFL
                * *    * *:   : ..:               :  :.                  * :

SSO1991         PPYPIIPGR-----------ITLILPGLIREGEEYTQVPDDNYFISKVKERYNEGWRKLI
Hbut_0714       PCIDEDDYERLRLEAEKLKTVSDDVFKLLEALRSCDGEKLRKYFIGRFWEGWRKVWCAAI
Igni_0328       PACSIELMR---------SELVEKGSLKEGEGVSDPSVIISRYFKERLKEAWKNVIEDIQ
Igni_0463       PRCAIELMK---------SYLEDK---HPKDLKLDSTKIIKMYFETRLREAWEDFVKKLL
                *       .           :                     **  :. * :..

SSO1991         EGLKCYSELKREDGFWNLVCR---------VLKLTEDLLQTTPLNIRVKQVSVTKDEIFN
Hbut_0714       ESARVGRGCNIEKFAETIVDLGVTDLSPEAVREYLEASRNNPPMQLRIVVINIEDEYDRL
Igni_0328       TRLRSTADNLIKTLEKVRVCT--------GTCTDVASADDLIERMIEIIKHAERAPFDVK
Igni_0463       EGLNSVGEAGLEGLRTFGAEG--------ELGAVLERVEDKPPFEVRVTVIDLRKALTEF
                   .       :      .                    :     :.:

SSO1991         NSKLRSDS----WKIYDNKYRQLVSEFK------KSKLVKVTPESRLKLFELTKFDKLPQ
Hbut_0714       LGWLREKKGKLSSLRVEEAAQKLLFTWLFTRALPEAEEREYAKSVSIDAGFAIAESLEEA
Igni_0328       TVVINLEE---AFEEFVKEFRRIEGKMR----AELEQLISSEGLAVGKDVFPYSKDLSDV
Igni_0463       NKVLKERS---LIRGVKDELRELLAKKYRVRVGPGEDLVKVIKGKVSDKVLPYTRNENDL
                   :.  .         .  :.:             .  .       .       .

SSO1991         IGEKSKRGYEFCTSCGVLPAVIIMPKEDEFEKKLIELGIARDEKDVRSIKNMISPGERLC
Hbut_0714       TSKPLKPEFHECSMCGRLPAIVHLADAAKVREFAER---LGVPV-----VLFSEGES-LC
Igni_0328       MSDLEKKLFFHWLVTKKYPEEFRKAKAVRLDPLLLDGWTLEVTK-----ARYESCKDKSA
Igni_0463       ERDLERKLFFHWLVTSKH-EAVKGAEDVGVDPRLVDGWSLEEGK-----RTYESCVR-NR
                  .  :  :            .  ..   .                       .

SSO1991         PWCLVKRALGAEPRLMRILLLGDLCSVEKIVNEIVSKDVKIEIP-----STSDIASIKTF
Hbut_0714       PYCLVRRLVSTSDAIRRIMNGLNLYYSLNPRQLYTRPPSTDELA----AMDKLLAIVDAL
Igni_0328       PMCTCGRPAAVHNPSEASSPLLRSHEALCPYCLILRLLQYYTDALTSIVEADHVKAPKVV
Igni_0463       PLCACGRPAAVHNPTDKDTKFVRAHEALCPYCLALRLAQHVP-------LAEGLRAPRAL
                * *   *  ..                                        . :    ..
```

```
SSO1991         EEMIEKKNEICEDLKEEEVCEKPNESMLSMWQRFNKNYYTGINLTIDPEEYWFSEKRRRY
Hbut_0714       SKDEQLAAELRSFFCKDDNGRNACEYRGKYVSEIVERYAYTKAGQKAKEILGLLEAIYDK
Igni_0328       MSTLAALPELVKWLSESGGA-----------VEIMDENGNNVGLPKDQLLESLTRSLKEY
Igni_0463       TSTLAAAPELACWLIEKG-------------------KASAKGARGRELVWTLARALLEP
                 .      *:   : :.                                   : .

SSO1991         YFSLFR----------------RHRITFPSPYYALVRADSDYLGDLLEGKLTPYLAGIID
Hbut_0714       LLGEFGEEFPHMLIAGILASEQRCASHLAKSVAKIREKREERVGSEVQEKAREVCSLLAK
Igni_0328       SSFDF--------------YQNKYSRFSLEEVLEELQ-KNETVERLIRDDSKNLYIRVLV
Igni_0463       P--------------------PGATRLNLEALPEELEEKGELVAGLEG-----FVIRITK
                                             .      .   : :              :

SSO1991         SGDYANISE-KKEEVNKLLEEYLVNAGSGPIVDYVKTVLECIRGNLNKCSCAVKIYSNEV
Hbut_0714       LYSRADG---VAGRYYAIVRGDGDYFGSRIIKGVLDFKSSRDYVEEMLKAIRDEGARSRL
Igni_0328       RKDRDSSKLLSLNKYIAMVKADADNMGN-LKGGRLGYDAEAYFETIYRQAGPRRGVGQEE
Igni_0463       EDLRAIN---SMNKYLAVIKSTVDRLDD-LKKGRLPYGTEEYFR-----EAVKKGTGSEG
                             .   ::.      ..    . :    .             .   ..

SSO1991         AEVMFRANRLLRKKLEKIDVEREVENSLKYFRTILKEGRIIVTPAWHVSISSALNRGLLV
Hbut_0714       APVYSEIATLLVDLMEEMKPETPSSGCKKGCKALPK-PATLVTPTYYMALSRGQMITALY
Igni_0328       -KLYKLAGSLVRSVIERLHEIYPKEVY--GENDSSL-PTVLVTPTYLFQLSYSLMTEALV
Igni_0463       GKDLELAIDIIKIVVDFARGLAPQRAG--GGEAG---QTVLVTPTYLSQLSYSLMTQALV
                         ::   ::               .        :***::   :* .     *

SSO1991         ELELINKHKGFVIYAGGDDLLAMLPV---------------------DEVLDFVKESRR-
Hbut_0714       DAEIVAMLGGFPVYAGGDDVAALAPG---YISKGRLENLIKGYATRATHIKDSIRADTGF
Igni_0328       DKEIVEKNYGLLVFAGGDDLLALVPARSVSRGSGRAEPLGGLEEFLSRELLEIVKEFYFS
Igni_0463       DAKLIELNFGLPALASGGELVALVPARTRCGPKGLPEELG--KLLKDETLLKRVKEDYYS
                : :::    *:   *.*.:: *: *                        : . ::

SSO1991         ----AFAGVSTGRLGNMCLENGFARINN-AYYPSLPIVGRSYSVIIAHYADPLFFVINDS
Hbut_0714       VPALIALYTRKNYWGLLWAGRGFHRTPIGAVYPAPVAYGRSYGIYIVHYRDPFMAAWRSA
Igni_0328       PALWVWWLTRLNHWGLLRSPVGFRYTDN-FFAPALLAYGRSYGIAIRHYRDPLAKVFEDA
Igni_0463       PALWAWWLTRLNHWGLLGEPKGFRRSEL-FFAPALLAYGRRYGIAIRHFKDPLAKVYEEA
                        .  .  * :  **           *:    ** *.: * *: **:  . ..:

SSO1991         YNLLEEGKEMIRYRVMYNGEYKDAKKDVAIFRYQGLT------SVIPLSLKRPIVNSVSD
Hbut_0714       GDLEEYK-DVIAFTSPHGTTVSKDATFLAYGRVSSIAGVELGAVALPNMKPGAGKEKVIT
Igni_0328       SELEES---------AKNVSKKKDGVGVSYGRLGARG------VALSNSLGVEDKGDLKE
Igni_0463       EGLEAP---------LRSTG---DWTGVSYGRLGAEG------VASPNSLGASRKEEVAE
                  *              .          ::  *  .          . .         .:

SSO1991         FNEIASIIDLILELKKR------------IDEGHISVSLLYDYEEYKHLI----------
Hbut_0714       WGAAGRKPEEPEPVGWTLALSLDLASKVEEEDRVISRSLYSDFERECPLAMRIARKIVLT
Igni_0328       PASLGPIIAKLSSFHR----------------RGLSNNFYYDIVRELQKYFGHDAKHEWA
Igni_0463       RG-LGPTTASLAFLNY----------------KGLSNNYYYDLISFVGKY---------A
                    .        .                    :* .   *

SSO1991         VASDEKYLTEFLVKDWIKRNSLRKH-VEFTIDEKLYGVRLTIENYPIKIPNDLISNIVYT
Hbut_0714       GSTDLQRIAESLLEMIIRRNTPERHRRKIEYITKLLKEVAMVAPAPAKLLEALECGQLRV
Igni_0328       AAKGLTTPIIILLKYIISRNVADEEKYSQNLKKEVEEVLEFLKLTNEYIFDVLEFGFAWH
Igni_0463       SAGGSSEALEALIEYVLKRNVVSGG--EGAAKEALGRLKRALGAGAEGLEHLLEFGFHWH
                 : .       *::  : **       .      :       :      : . *  .

SSO1991         LRIIYGGEK--------------------------
Hbut_0714       EEAEKRLNGFSKLACLAPLPWQIVLAAYTISSGRR
Igni_0328       KAVR-------------------------------
Igni_0463       KAAR-------------------------------
```
