# Supplementary material for: Gain and loss of an intron in a protein-coding gene in Archaea: the case of an archaeal RNA pseudouridine synthase gene
Source: BMC Evol Biol. 2009 Aug 11;9:198. doi: 10.1186/1471-2148-9-198 (PMC2738675; doi:10.1186/1471-2148-9-198)
Supplement: Additional file 10 — Figure legends for Additional files. Legends for Additional files 3, 4, 6, 8 and 9 are shown. [file 1471-2148-9-198-S10.pdf]

Additional file 10. Figure legends for supplementary figures.

Additional file 3. Alignment of archaeal Cbf5 sequences used in the analysis for Figure S2.

#; selected positions for the analysis.

Additional file 4. Bayesian phylogenetic tree of crenarchaeal Cbf5 protein.

Posterior probability (PP) and bootstrap probability (BP: %) are shown at the nodes. See sequence details in Table 3, except for APKG8O8 (uncultured marine crenarchaeote HF4000_APKG8O8, accession number [Genbank:ABZ09863]), APKG5E24 (uncultured marine crenarchaeote HF4000_APKG5E24, [Genbank:ABZ08853]), APKG8G2 (uncultured marine crenarchaeote HF4000_APKG8G2, [Genbank:ABZ09625]) as well as *Methanocaldococcus jannaschii* [Genbank:AAB98132], '*Nanoarchaeum equitans*' [Genbank:AAR39298)], and *Methanopyrus kandleri* [Genbank:AAM01350] as the outgroups. Species with the *cbf5* intron are shown with underlined letters. Bold lines show lineages with the *cbf5* intron.

Additional file 6. Alignment of archaeal Cbf5 sequences used in the analysis for Figure 2.

#; selected positions for the analysis.

Additional file 8. Bayesian phylogenetic tree of the crenarchaeal 16S rRNA.

Posterior probability (PP) and bootstrap probability (BP: %) are shown at the nodes, respectively. Sequences from *Nanoarchaeum equitans*, *Metahanocaldococcus jannaschii*, *Methanopyrus kandleri*, and *Candidatus* Korarchaeum cryptofilum are added as the outgroups. Species with the *cbf5* intron are shown with underlined letters. Bold lines show lineages with the *cbf5* intron.

Additional file 9. Alignment of COG1353 proteins.

Sulfolobus solfataricus SSO1991 [Genbank:NP_343391.1], a representative of COG1353 which was predicted as a putative reverse transcriptase [1], and the homologs from *Hyperthermus butylicus* (Hbut_0714, [Genbank:YP_001012914.1]), and *Ignicoccus hospitalis* (Igni_0328

[Genbank:YP_001434918.1] and Igni_0463 [Genbank:YP_001435053.1]) are included. The alignment was performed with Clustal X [2].

References

1. Makarova KS, Grishin NV, Shabalina SA, Wolf YI, Koonin EV: **A putative RNA-interference-based immune system in prokaryotes: computational analysis of the predicted enzymatic machinery, functional analogies with eukaryotic RNAi, and hypothetical mechanisms of action.** *Biol. Direct* 2006, **1**:7.

2. Thompson JD, Higgins DG, Gibson TJ: **CLUSTAL W: improving the sensitivity of progressive multiple sequence alignment through sequence weighting, position-specific gap penalties and weight matrix choice.** *Nucleic Acids Res* 1994, **22**:4673-4680.
